# Design and Implementation of Observational Studies Emulating a Target Trial

**DOI:** 10.1001/jamanetworkopen.2025.58262

**Published:** 2026-02-19

**Authors:** Yan Ren, Yulong Jia, Lingyan Liu, Houchen Lyv, Liyuan Tao, Yuezhen Li, Peng Zhao, Yiquan Xiong, Guowei Li, Jiayue Xu, Chunrong Liu, Yongmei Lu, Kang Zou, Jing Tan, Xin Sun

**Affiliations:** 1 General Practice Medical Center, Chinese Evidence-Based Medicine Center, West China Hospital, Sichuan University, Chengdu, China; 2 National Health Commission Key Laboratory of Clinical Epidemiology and Evidence-Based Medicine, West China Hospital, Sichuan University, Chengdu, China; 3 Sichuan Centre of Technology Innovation for Real World Data, West China Hospital, Sichuan University, Chengdu, China; 4 Department of Orthopaedics, Chinese People’s Liberation Army General Hospital, Beijing, China; 5 Research Center of Clinical Epidemiology, Peking University Third Hospital, Beijing, China; 6 Medical Examination Centre, Peking University Third Hospital, Beijing, China; 7 The Affiliated Guangdong Second Provincial General Hospital of Jinan University, Guangzhou, China; 8 Department of Epidemiology and Biostatistics, West China School of Public Health, Sichuan University, Chengdu, China

## Abstract

**Importance:**

Target trial emulation (TTE) is increasingly used to estimate causal effects with observational data, yet its clinical applications and methodologic quality remain unclear.

**Objective:**

To characterize the clinical scenarios where TTE has been applied, assess methodologic limitations in design and implementation, and provide structured recommendations for strengthening future practice.

**Design, Setting, and Participants:**

This cross-sectional study searched PubMed for TTE studies published from January 1, 2017, to December 15, 2023, in clinical journals in the top Journal Impact Factor quartile, supplemented by studies from 3 prior scoping reviews. Eligible studies explicitly aimed to emulate a target trial using observational data. Data were extracted using a standardized, consensus-based tool.

**Main Outcomes and Measures:**

Study characteristics, application scenarios, specification of target trials, and adherence to 7 methodologic domains of TTE were evaluated. Recommendations were developed to address observed gaps.

**Results:**

Among 237 included studies, over half (128 [54.0%]) evaluated drug interventions, commonly in infectious diseases, cardiology, and oncology. TTE was applied in 8 recurring clinical scenarios, including randomized clinical trial replication and extending evidence to underrepresented populations or rare outcomes. TTE was most frequently applied to assess treatment effectiveness (165 studies [69.6%]), long-term outcomes (39 [16.5%]), emergency-use medications (42 [17.7%]), and active-treatment comparisons (49 [20.7%]). Major methodologic gaps were evident: only 134 studies (56.5%) developed a prespecified protocol, 127 (53.6%) did not review existing randomized clinical trials, and most that emulated existing trials (20 of 31 [64.5%]) or hypothetical trials (147 of 153 [96.1%]) did not justify the choice of trial to emulate. During implementation, 103 studies (43.5%) did not report all 7 methodologic components, 36 (15.2%) used postbaseline information inappropriately for eligibility, only 40 (16.9%) provided follow-up diagrams to define time zero, and only 73 (30.8%) addressed unmeasured confounding. A 5-step framework for target trial design and practical considerations for implementing methodologic principles was developed.

**Conclusions and Relevance:**

This cross-sectional study of TTE identified 8 common clinical scenarios for TTE application and substantial methodologic heterogeneity that threatened the credibility of resulting evidence. The study highlighted common pitfalls and proposed actionable recommendations to complement reporting standards such as the Transparent Reporting of Observational Studies Emulating a Target Trial guideline, which may support the generation of more robust observational evidence for clinical and policy decision-making.

## Key Points

**Question:**

In which clinical scenarios has target trial emulation (TTE) been applied, and how well do TTE studies adhere to key principles of target trial design and implementation?

**Findings:**

In this cross-sectional analysis of 237 TTE studies (2017-2023), 8 recurring clinical scenarios and substantial variation in design quality and methodologic implementation were identified. A structured framework and practical recommendations were proposed to address these gaps.

**Meaning:**

These findings highlight the need to improve methodologic rigor in TTE, and the provided recommendations complement the Transparent Reporting of Observational Studies Emulating a Target Trial guideline by offering practical, scenario-based guidance for strengthening TTE methods.

## Teaser

This cross-sectional analysis of studies using target trial emulation assesses clinical scenarios in which this approach has been applied, identifies common methodologic challenges, and proposes a structured framework for rigorous target trial design.

## Introduction

Target trial emulation (TTE) is an increasingly adopted framework for estimating causal effects using observational data.^1,2^ By explicitly mimicking a hypothetical randomized clinical trial (RCT), TTE aims to reduce bias and enhance the credibility of observational studies.^3,4,5,6^ The methodologic foundation was established by Hernán and Robins,^7,8^ and the empirical validity from the RCT DUPLICATE initiative^9^ has accelerated the uptake of TTE across diverse domains, including infectious diseases, cancer, and cardiovascular research.^10,11,12,13,14,15,16^

Despite the growing popularity of TTE, important uncertainties remain. It is not fully understood in which clinical scenarios TTE has been applied or whether such applications are always conceptually appropriate. A distinguishing feature of TTE, in contrast to traditional observational studies, is the emphasis on explicitly defining a target trial aligned with the research purpose.^17^ However, methodologic guidance on how to design such trials remains underdeveloped.

Several efforts have contributed to improving the conduct and reporting of TTE. Methodologic studies have addressed specific challenges, such as avoiding immortal time bias,^18,19^ selecting appropriate comparators,^20^ or implementing sustained treatment strategies through techniques such as cloning or censoring.^21,22,23,24,25,26^ Previous reviews have also examined reporting quality, specific biases, clinical disease areas, or specific components of TTE implementation.^27,28,29,30,31^ Most recently, the Transparent Reporting of Observational Studies Emulating a Target Trial (TARGET) reporting guideline introduced a structured checklist to enhance reporting clarity and reproducibility.^32^ However, TARGET focuses on what should be reported rather than how target trials should be designed or technically executed. Likewise, existing reviews do not offer context-specific, practice-oriented recommendations for designing and implementing TTE studies across diverse clinical scenarios.

To address these gaps, we conducted a cross-sectional methodologic survey of published TTE studies. Our objectives were to (1) characterize the clinical scenarios in which TTE has been applied, (2) evaluate the specification and implementation of target trials, and (3) identify common methodologic challenges. Building on these findings, we propose a structured, scenario-based framework for target trial design and provide practical recommendations for applying the 7 core methodologic elements essential for rigorous TTE.

## Methods

The protocol of this cross-sectional study was registered on the Open Science Framework website.^33^ Given that the unit of analysis comprised published studies, ethics approval and informed consent were not applicable. We followed the Strengthening the Reporting of Observational Studies in Epidemiology (STROBE) reporting guideline.

### Data Sources

We systematically searched PubMed to identify TTE studies published between January 1, 2017, and December 15, 2023. To ensure quality, we restricted the search to studies published in Journal Impact Factor quartile 1 journals under the “clinical medicine” category of the Journal Citation Reports. We used a combination of MeSH terms and keywords related to TTE, such as target* trial*, emulat* trial*, and trial* emulat*, to search for potentially eligible studies (eAppendix 1 in Supplement 1). To ensure comprehensiveness, we also screened eligible studies from 3 previously published scoping and systematic reviews on TTE.^27,28,29^

### Eligibility Criteria

We included original studies that explicitly stated the use of “target trial emulation” or clearly indicated the emulation of a trial using observational data. We excluded studies that met any of the following criteria: (1) case report or series, editorial, letter, commentary, study protocol, abstract, systematic review, meta-analysis, or randomized controlled study; (2) not published in English; (3) methodologic study discussing TTE without applying it; or (4) only mentioned the concept of trial emulation—such as in sensitivity analyses or as part of confounding control—without explicitly identifying the study as a TTE.

### Study Selection

We used a predesigned, machine learning–assisted framework—fuzzy population–intervention study design (fPIS) combined with gradient boosting decision tree ensemble modeling—to assist in identifying eligible TTE studies (eAppendix 2 in Supplement 1). The algorithm analyzed full-text content to classify articles as “yes” (a TTE study) or “no” (not a TTE study). Two researchers (Y.R., Y.J.) trained in clinical epidemiology and TTE independently screened full texts for studies classified as “yes” and a randomly selected subset of “no” classifications to minimize false negatives. Disagreements were resolved through discussion or, if needed, adjudication by a third investigator (J.T.).

### Questionnaire Development and Data Extraction

We developed a standardized data extraction form based on relevant methodologic literature.^3,4,7,8,27,28,29,34,35,36,37^ The initial draft was created by 2 researchers (Y.R., Y.J.) and reviewed by a panel of 6 domain experts (H.L., L.T., Y.X., J.X., J.T., X.S.). The form was refined following pilot testing on 20 studies and consensus discussion. The final form (eAppendix 3 in Supplement 1) included 5 domains: general study characteristic; population, intervention, control, and outcome (PICO) elements; clinical scenarios; target trial design characteristics; and implementation of core methodologic components.

All investigators (Y.R., Y.J., L.L., Yuezhen L., P.Z., Y.X.) were trained on the definitions and procedures. To ensure consistency, each investigator conducted a pilot extraction on 5 studies, with discrepancies discussed as a group. Each article was independently extracted by 1 reviewer (Y.J., Yuezhen L., P.Z., or Y.X.) and verified by a second reviewer (Y.R., Y.J., or L.L.) with expertise. If multiple TTEs were presented within a single article, we collected information regarding only the first reported emulation; if several target trials were emulated within a single article, we collected information regarding only the first reported target trial.

### Recommendations Development

We designed a structured questionnaire and conducted an expert consensus process to develop recommendations for the design and implementation of TTE studies. First, we generated candidate recommendations based on the findings from our survey of methods in TTE research and related methodologic studies. Next, we designed a structured consultation questionnaire (eAppendix 4 in Supplement 1). Then, we invited 22 experts from multidisciplinary fields—including TTE, epidemiology, statistics, clinical trials, and clinical medicine—who had experience conducting observational studies or TTE research to participate in the expert consensus process. Subsequently, 6 additional external experts were invited to independently evaluate the scenario-based framework.

### Statistical Analysis

Descriptive analysis was used to summarize the study characteristics. Categorical variables were presented as frequencies and percentages. Continuous variables were reported as means with SDs or medians with IQRs, as appropriate. All analyses were conducted using SAS software, version 9.4 (SAS Institute Inc).

## Results

A total of 46 986 records were identified through the PubMed search. After automated screening using the fPIS–machine learning technique, 46 024 records were excluded. The remaining 962 records underwent manual eligibility assessment. An additional 334 records were retrieved from the 3 previous scoping reviews on TTE.^27,28,29^ In total, 237 records met the eligibility criteria and were included in the final analysis^6,10,15,19,21,38,39,40,41,42,43,44,45,46,47,48,49,50,51,52,53,54,55,56,57,58,59,60,61,62,63,64,65,66,67,68,69,70,71,72,73,74,75,76,77,78,79,80,81,82,83,84,85,86,87,88,89,90,91,92,93,94,95,96,97,98,99,100,101,102,103,104,105,106,107,108,109,110,111,112,113,114,115,116,117,118,119,120,121,122,123,124,125,126,127,128,129,130,131,132,133,134,135,136,137,138,139,140,141,142,143,144,145,146,147,148,149,150,151,152,153,154,155,156,157,158,159,160,161,162,163,164,165,166,167,168,169,170,171,172,173,174,175,176,177,178,179,180,181,182,183,184,185,186,187,188,189,190,191,192,193,194,195,196,197,198,199,200,201,202,203,204,205,206,207,208,209,210,211,212,213,214,215,216,217,218,219,220,221,222,223,224,225,226,227,228,229,230,231,232,233,234,235,236,237,238,239,240,241,242,243,244,245,246,247,248,249,250,251,252,253,254,255,256,257,258,259,260,261,262,263,264,265,266,267,268,269^ (eFigure 1 in Supplement 1).

The expert panel consisted of 22 multidisciplinary specialists from 4 countries, primarily in fields of epidemiology and statistics. Eleven (50.0%) held senior academic titles, 5 (22.7%) had published TTE studies, and 11 (50.0%) had participated in TTE-related research. All panelists were familiar with TTE methods.

### General Characteristics

The number of published TTE studies increased steadily from 2017 to 2023, with a marked rise beginning in 2021 (eFigure 2 in Supplement 1). Corresponding authors were most commonly based in the US (98 [41.4%]). As shown in eTable 1 in Supplement 1, most studies used a retrospective cohort design (207 [86.4%]), and nearly half (118 [49.8%]) relied on electronic medical records. National-level data sources were used in 175 studies (73.8%). The median analyzed sample size was 11 200 (IQR, 2381-106 112). Methodologists were involved in 193 studies (81.4%), and 197 (83.1%) reported receiving financial support.

### PICO Elements

As shown in Table 1, the most common disease areas were infectious diseases (59 studies [24.9%]), cardiology (33 [13.9%]), and oncology (25 [10.5%]). More than half of the studies (128 [54.0%]) evaluated drug interventions. In comparator selection, 119 studies (50.2%) used active controls and 38 (16.0%) used standard care. All-cause mortality was the most frequently examined primary outcome (102 studies [43.0%]). Most outcomes (153 studies [64.6%]) were measured on a time-to-event scale.

**Table 1.** PICO Characteristics of Included TTE Studies

Variable	Studies, No. (%) (N = 237)
Target disease	
Infectious diseases (COVID-19, HIV, and others)	59 (24.9)
Cardiology (cardiovascular diseases)	33 (13.9)
Oncology	25 (10.5)
Endocrinology (diabetes and other diseases)	19 (8.0)
Neurology (cerebrovascular diseases)	6 (2.5)
Rheumatology	9 (3.8)
Psychiatry	7 (3.0)
Kidney disease	9 (3.8)
Orthopedic diseases	5 (2.1)
Obstetrics and gynecology	5 (2.1)
Critical care medicine	5 (2.1)
Other	23 (9.7)
No specified disease	32 (13.5)
Type of intervention	
Drug	128 (54.0)
Vaccine	25 (10.5)
Surgical	21 (8.9)
Behavioral	16 (6.8)
Other^a^	47 (19.8)
Type of control^b^	
Standard care	38 (16.0)
Blank control	93 (39.2)
Active control	119 (50.2)
Other^c^	4 (1.7)
Primary outcome^b^	
All-cause mortality	102 (43.0)
Disease-specific mortality	19 (8.0)
Cardiovascular major morbid events	38 (16.0)
Other major morbid events (eg, loss of vision, seizures, fracture, revascularization)	53 (22.4)
Onset, recurrence, relapse, and/or remission of cancer and other chronic diseases (eg, COPD exacerbation)	21 (8.9)
Hospitalization	31 (13.1)
Infections	24 (10.1)
Symptoms, quality of life, and functional status	18 (7.6)
Laboratory examination	16 (6.8)
Other^d^	6 (2.5)
Type of primary outcome	
Continuous	12 (5.1)
Ordinal	1 (0.4)
Binary	72 (30.4)
Time-to-event	153 (64.6)
Other^e^	3 (1.3)

Abbreviations: COPD, chronic obstructive pulmonary disease; PICO, population, intervention, control, and outcome; TTE, target trial emulation.

^a^ Included medical devices, invasive nonsurgical procedures, psychological intervention, rehabilitation, medical examination, and others.

^b^ Some studies had more than 1 option.

^c^ Included dose response and different change trajectories of medical indicators.

^d^ Included patient adherence, outpatient visits, and use of antibiotics.

^e^ Included count data and ratios.

### Objectives and Application Scenarios

Most studies (165 [69.6%]) aimed to evaluate treatment effectiveness. We identified 8 clinical scenarios in which TTE was applied. The application scenarios addressed were heterogenous. A minority of studies focused on broader populations beyond RCT eligibility (21 [8.9%]) or on rare diseases (4 [1.7%]). Regarding intervention contexts, 42 studies (17.7%) evaluated emergency-use medications (eg, COVID-19 treatments), 49 (20.7%) assessed active-treatment comparisons, and 5 (2.1%) focused on treatment switching. For outcomes, 39 studies (16.5%) examined long-term effects and 8 (3.4%) evaluated rare-event outcomes. Additionally, 46 (19.4%) addressed settings with conflicting prior evidence, 35 (14.8%) examined questions lacking RCT evidence, and 19 (8.0%) estimated the results in actual clinical settings, while 22 (9.2%) attempted RCT replication and 5 (2.1%) aimed to predict future trial results (Table 2).

**Table 2.** Clinical Characteristics of Included TTE Studies

Variable	Studies, No. (%) (N = 237)
What did the study aim to evaluate?	
Effectiveness	165 (69.6)
Safety	56 (23.6)
Safety and effectiveness	15 (6.3)
Cost-effectiveness	1 (0.4)
Which scenario did the emulated trial present?^a^	
Replicating the results of a published trial in an actual clinical setting	22 (9.2)
Predicting the results of the RCTs in advance	5 (2.1)
Estimating the previous conflicting results reported in studies	46 (19.4)
Estimating the effectiveness or safety of emergency medication (eg, COVID-19 drug)	42 (17.7)
Estimating the comparative effectiveness vs active control	49 (20.7)
Estimating the effectiveness of treatment switching	5 (2.1)
Estimating the effectiveness or safety for a long-term outcome	39 (16.5)
Estimating the safety for a rare-event outcome	8 (3.4)
Estimating the effectiveness or safety for rare diseases	4 (1.7)
Estimating the effectiveness or safety for the broader population	21 (8.9)
Lacking RCT evidence	35 (14.8)
Estimating the effectiveness or safety in an actual clinical setting	19 (8.0)
Other^b^	20 (8.4)
What types of treatment strategies did the target trial emulation focus on?	
Point treatment strategies	98 (41.4)
Static sustained treatment strategies	128 (54.0)
Dynamic sustained treatment strategies	11 (4.6)
What types of treatment strategies did the target trial emulation compare?^a^	
Head-to-head comparison of ≥2 active treatments (A vs B)	125 (52.7)
Single active treatment vs no treatment (A vs none)	98 (41.4)
Combination treatment vs 1 active treatment (A + B vs A)	18 (7.6)
Combination treatment vs no treatment (A + B vs none)	2 (0.8)
Other^c^	10 (4.2)

Abbreviations: RCT, randomized clinical trial; TTE, target trial emulation.

^a^ Some studies included more than 1 option.

^b^ Included use of published data to answer different problems or a primary outcome that included count data or ratios.

^c^ Included combination treatment vs combination treatment, high dose vs low or standard dose, and different laboratory indicator levels.

Over half of the studies (128 [54.0%]) evaluated static sustained treatment strategies, 98 (41.4%) examined point treatment strategies, and 11 (4.6%) examined dynamic strategies. Treatment comparisons frequently involved head-to-head comparisons (125 [52.7%]) and a single active treatment compared with no treatment (98 [41.4%]).

### Methodologic Characteristics of Target Trial Design

As shown in Table 3, only 40 studies (16.9%) reported prospective protocol registration, and 134 (56.5%) explicitly developed a target trial protocol. More than half (127 [53.6%]) did not review existing RCTs during trial specification. Among the included studies, 31 (13.1%) emulated existing RCTs, while 153 (64.6%) emulated hypothetical trials; however, most of those (20 of 31 [64.5%] and 147 of 153 [96.1%], respectively) did not justify their choice of trial to emulate. Twenty-seven studies (11.4%) reported trials with more than 2 arms, and 37 studies (15.7%) emulated 2 or more target trials, primarily due to variations in interventions or populations.

**Table 3.** Methodology Characteristics for Designing a Target Trial in the Included TTE Studies

Variable	Studies, No. (%) (N = 237)
Has the research protocol been registered?	
Yes	40 (16.9)
No	197 (83.1)
Was a target trial protocol made?	
Yes	134 (56.5)
No	103 (43.5)
How many target trials were emulated in this study?	
1	175 (73.8)
2	21 (8.9)
≥3 (please specify)	16 (6.8)
Not reported	25 (10.5)
If ≥1 target trial was conducted, what was the reason?	
Different intervention, No. /total No. (%)	18/37 (48.6)
Different primary outcome, No. /total No. (%)	1/37 (2.7)
Different population, No. /total No. (%)	14/37 (37.8)
Other, No. /total No. (%)	6/37 (16.2)
How many arms were compared in target trials?	
1	8 (3.4)
2	162 (68.4)
3	13 (5.5)
4	6 (2.5)
>4	8 (3.4)
Not reported	40 (16.9)
Did the researcher review existing RCTs for the target trial?	
Yes	110 (46.4)
No	127 (53.6)
Which type of target trial was the study planned to emulate?	
Existing complete RCT	26 (11.0)
Existing ongoing RCT	5 (2.1)
Hypothetical RCT	105 (44.3)
May be a hypothetical RCT	48 (20.3)
Not specified	53 (22.4)
If the emulated trial was an existing RCT, did the researcher give the reason for selecting this RCT as the target trial?	
Yes, No./total No. (%)	11/31 (35.5)
No, No./total No. (%)	20/31 (64.5)
If the emulated trial was a hypothetical RCT, did the researcher give the reference standards for defining such a trial?	
Yes, No./total No. (%)	6/153 (3.9)
No, No./total No. (%)	147/153 (96.1)
Which type of RCT was the study claimed to emulate?	
Parallel RCT	33 (13.9)
Cluster RCT	4 (1.7)
Pragmatic trial	49 (20.7)
Other	4 (1.7)
Not specified	147 (62.0)

Abbreviations: RCT, randomized clinical trial; TTE, target trial emulation.

### Methodologic Characteristics of TTE Implementation

Overall, 103 studies (43.5%) did not report all 7 core components of TTE (eTable 2 in Supplement 1). Inconsistencies between the emulated and target trials were reported in eligibility criteria (37 studies [15.6%]), treatment strategies (20 [8.4%]), and outcome definitions (10 [4.2%]). Thirty-six studies (15.2%) used postbaseline information inappropriately for eligibility determination, and 42 (17.7%) did not include flowcharts describing eligibility processes. Only 35 studies (14.8%) used a sequential design with multiple eligibility time points. Regarding treatment strategy design, 93 studies (39.2%) did not use active comparators, and 71 (30.0%) adopted new user design. Only 37 (15.6%) explicitly assessed the quality of the outcome.

Most studies (158 [66.7%]) reported efforts to emulate randomization, and 29 of those (18.4%) applied cloning techniques in scenarios where cloning was appropriate. Few studies (40 [16.9%]) provided follow-up diagrams to define time zero. Most studies (106 of 161 [65.8%]) described the causal contrast in the target trial as an intention-to-treat (ITT) analysis, while the emulated trial often used a per-protocol (PP) approach (eFigure 3 in Supplement 1). Although 185 studies (78.1%) conducted sensitivity analyses, only 73 (30.8%) used statistical methods to assess the impact of unmeasured confounding.

#### A Structured Framework for Designing Target Trials

**Figure 1.**
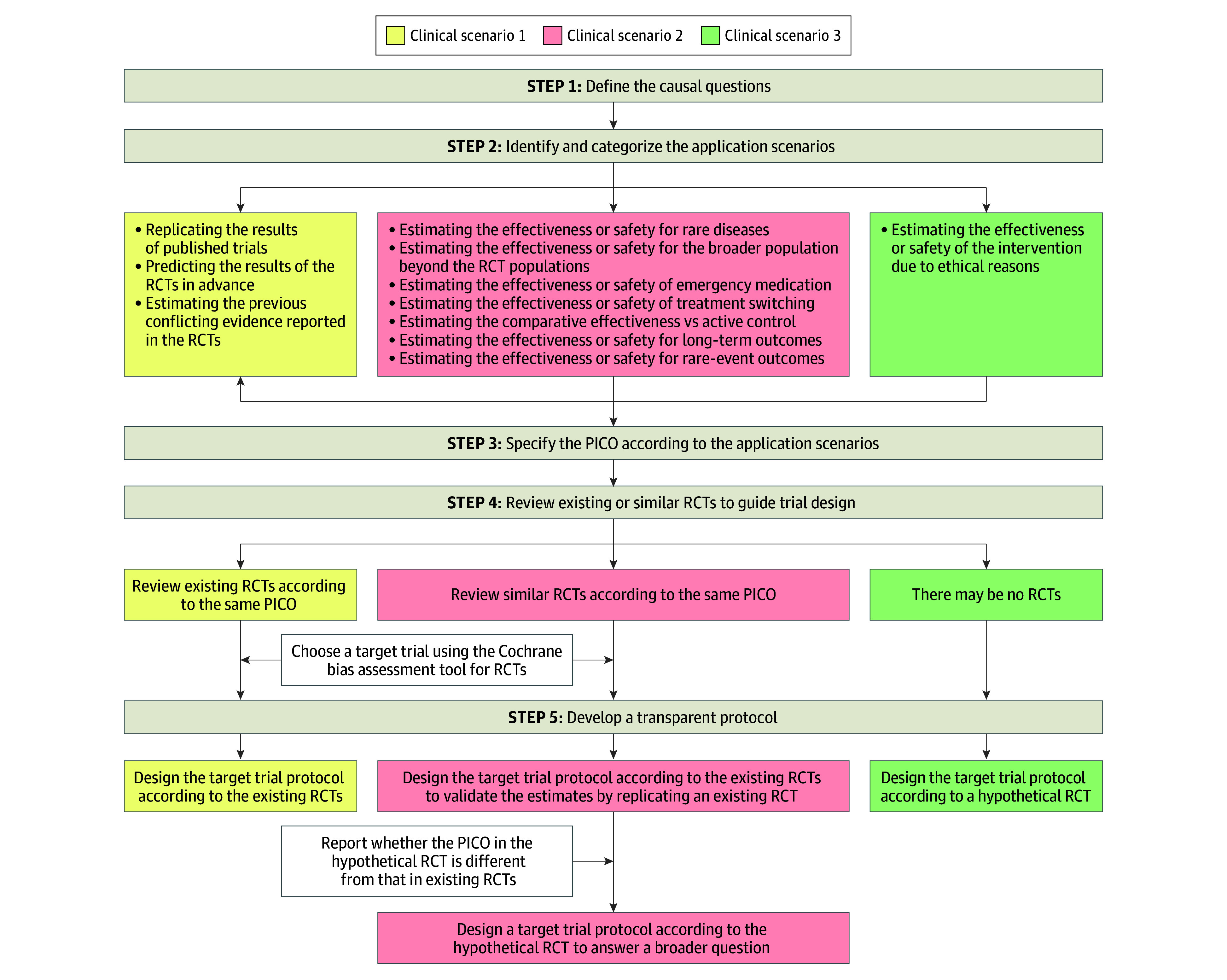
Diagram of the Recommended Framework to Design a Target Trial According to the Clinical Scenario PICO indicates population, intervention, control, and outcome; RCT, randomized clinical trial.

To enhance consistency and rigor in target trial specification, we developed a 5-step framework (Figure 1 and eAppendix 5 in Supplement 1). Step 1 is to define the causal questions and intervention effect of interest. Step 2 is to identify and categorize the application scenario into 3 types: (1) replication or prediction of RCT findings in clinical settings; (2) extension of evidence to new populations, rare outcomes, or treatment switching; or (3) settings where RCTs are infeasible due to ethical or logistic constraints. Step 3 is to specify all PICO elements to ensure alignment between purpose and design. Step 4 is to review existing or similar RCTs to guide trial design, with 3 scenarios: (1) emulate an existing RCT with matched PICO, (2) consult similar RCTs to inform a hypothetical trial specification, or (3) justify the design choices based on clinical reasoning when RCT evidence is absent. Step 5 is to develop a transparent protocol, which should explicitly describe all key components, including eligibility criteria, treatment strategies, assignment procedures, follow-up, outcomes, causal contrast, and the statistical analysis plan. For the second scenario in step 4, 2 protocols may be considered: 1 to replicate existing RCTs and 1 to address novel questions, as previously suggested.^6^

#### Considerations for Implementing TTE Methods

**Figure 2.**
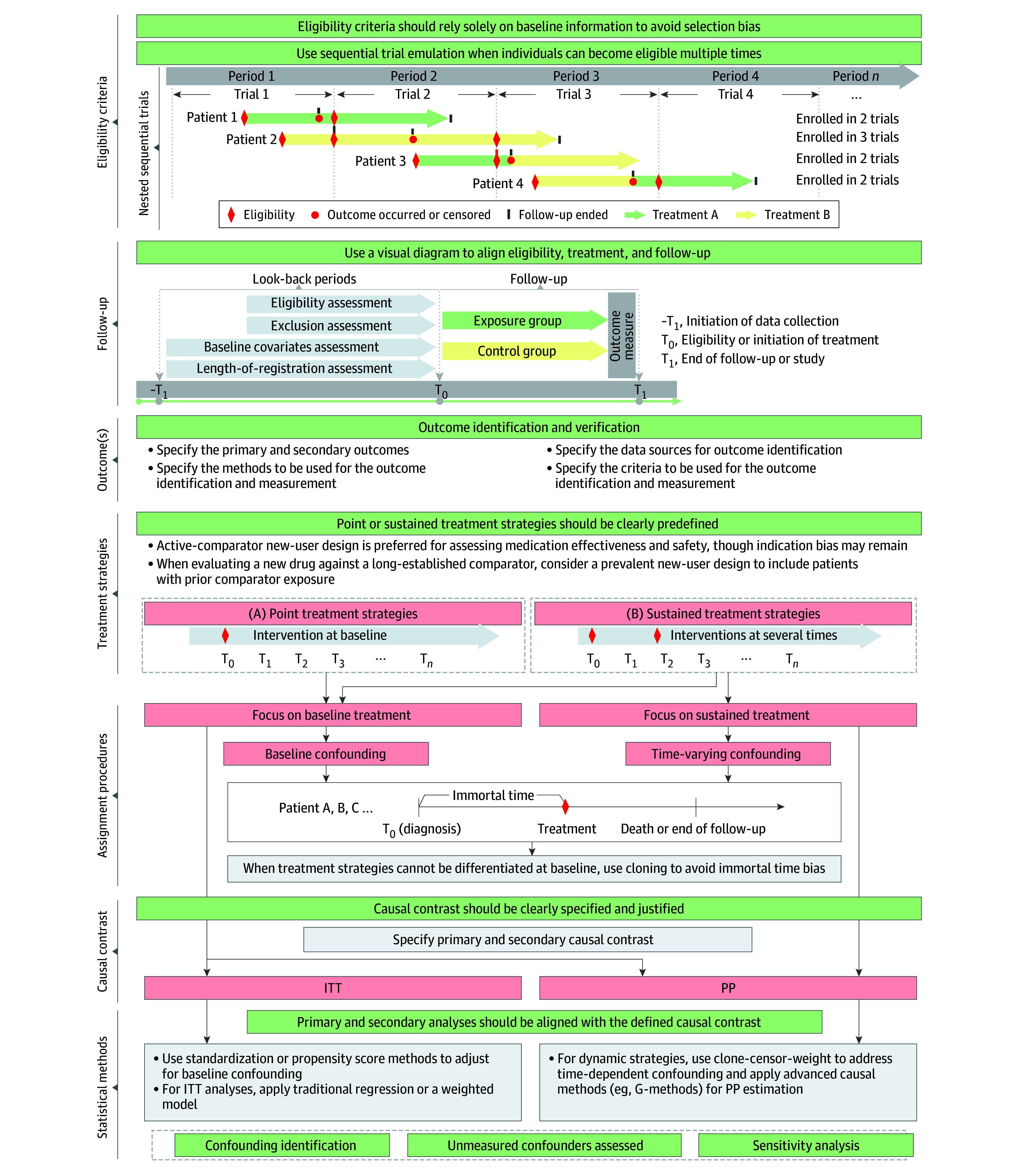
Diagram of the Methodologic Recommendation for Implementing a Target Trial Emulation Study ITT indicates intention to treat; PP, per protocol; T, time.

Drawing on identified gaps, we outline practical considerations for implementing the 7 core methodologic components of TTE (Figure 2 and eAppendix 5 in Supplement 1). First, eligibility criteria are generally expected to rely on information available at baseline to avoid selection bias. Sequential trial emulation is recommended when individuals become eligible multiple times during follow-up. Second, treatment strategies may benefit from adopting an active comparator new-user design. Point or sustained treatment strategies should be clearly predefined. Third, assignment procedures may be strengthened through analytic approaches (eg, propensity score matching, G-methods) to emulate randomization. Depending on the type of treatment strategy, different methods may be appropriate. Fourth, follow-up can be more clearly communicated using a visual design diagram that illustrates the alignment of eligibility, treatment initiation, and follow-up at time zero. Fifth, outcomes are ideally prespecified, with clear documentation of definitions, data sources, and any validation procedures to support outcome accuracy and relevance. Sixth, causal contrast (ITT or PP) should be clearly stated and contextually justified based on the research question and the type of treatment strategy. Seventh, statistical methods should be aligned with the defined causal contrast and might include techniques to explore or adjust for unmeasured confounding and sensitivity analyses to assess the robustness of findings. For dynamic treatment strategies, we recommend using the clone-censor-weight approach for time-dependent confounding and immortal time bias and using causal inference methods such as G-methods or doubly robust estimators to quantify treatment effects.

## Discussion

### Summary of Main Findings

In this survey of 237 published TTE studies, we observed a rapid increase in their use since 2021, which may reflect both the growing recognition of observational evidence and the substantial expansion of COVID-19–related research—consistent with prior findings that approximately 20% of TTE publications between 2020 and 2021 addressed COVID-19 questions.^29^ Most studies focused on pharmacologic treatment effectiveness and were conducted primarily in high-income countries using national or large-scale electronic health records. We identified 8 recurrent clinical scenarios in which TTE has been applied, ranging from RCT replication to extending evidence to underrepresented populations or rare outcomes. These findings suggest that TTE has become a versatile tool to complement RCTs, particularly in settings where trials are infeasible, underpowered, or ethically challenging. However, observed misuse of the framework in evaluating nonmodifiable exposures^117,171,242^ underscores the need for clearer conceptual boundaries.

Despite increasing adoption, major gaps remain in how target trials are designed. Fewer than 1 in 5 studies included in this cross-sectional survey prospectively registered a protocol, and nearly half did not explicitly develop a target trial protocol. More than half did not review existing RCTs before designing their target trial, and many failed to clarify whether they emulated a hypothetical or existing RCT. These shortcomings undermine transparency, reproducibility, and the interpretability of causal claims. Our proposed 5-step framework addresses these issues by emphasizing a priori protocol development, systematic use of the PICO framework, and explicit consideration of prior RCTs when defining the target trial.

The 7 core methodologic items of the TTE framework are key points for achieving causal inference through control bias.^36,242,270^ However, deviations from the 7 core methodologic elements of TTE were also common. Examples included the inappropriate use of postbaseline information for eligibility, inadequate description or implementation of procedures intended to emulate randomization, and inconsistent specification of time zero. Fewer than one-third of studies assessed the influence of unmeasured confounding, and fewer than one-fifth used visual diagrams to clarify follow-up design. These limitations pose risks of systematic bias and weaken causal inference, particularly in comparative effectiveness research. Strengthening TTE practice therefore requires both adherence to established principles (eg, active-comparator new-user designs) and broader adoption of causal inference methods, including cloning, censoring, and advanced sensitivity analyses.^35,54,271^

### Comparison With Other Studies

Previous methodologic reviews have examined reporting practices, specific biases, or selected clinical areas,^27,28,29,30,31^ although none provided a comprehensive evaluation across application scenarios or offered practice-oriented guidance for designing and implementing a TTE. Existing methodologic commentaries have proposed recommendations within specific contexts, such as diabetes, critical care, and rehabilitation,^272,273^ but they did not articulate a generalizable framework for target trial design.

The recently published TARGET guideline represents an important advancement by standardizing reporting for observational studies emulating a target trial.^32^ Its primary focus is on what should be reported to enhance clarity and reproducibility. However, TARGET does not offer technical guidance on how to specify or operationalize the target trial, nor does it provide scenario-specific recommendations for implementing TTE in practice.

In contrast, our study addressed gaps not covered by TARGET or prior reviews. Our 5-step framework and methodologic considerations are intended to guide the design and implementation of TTE studies before reporting occurs. The 5-step framework focuses on upstream design decisions—defining the causal question, identifying the clinical scenario, specifying PICO, reviewing existing or similar RCTs to guide trial design, and developing a transparent protocol. Our accompanying recommendations expand the 7 methodologic elements by offering operational, practice-oriented guidance for implementation, including handling multiple eligibility time points, selecting appropriate comparators, determining time zero, and applying cloning or censoring when warranted. Thus, TARGET and our recommendations play complementary but distinct roles: TARGET strengthens reporting, whereas our framework strengthens design quality and methodologic rigor, thereby supporting more credible and reproducible TTE research.

### Strengths and Limitations

This study has several strengths. We conducted a systematic evaluation of both clinical applications and methodologic rigor of TTE across a wide range of disease areas. The large and diverse sample enhances generalizability, and the integration of prior methodologic literature allowed us to develop practical, scenario-specific recommendations. Nonetheless, limitations should be noted. We included studies published between 2017 and 2023 in clinical journals with a Journal Impact Factor in quartile 1, which may not reflect the most recent developments or practices in lower-tier outlets. Our analysis relied on reported information, and unreported methodologic decisions could lead to underestimation of actual practice quality. In addition, we did not conduct a formal applicability assessment for the proposed recommendations.

## Conclusions

In this cross-sectional study, we documented the growing use of TTE for estimating causal effects in contexts where randomized trials are impractical, unethical, or unavailable. However, we identified substantial and persisting heterogeneity in how target trials were defined and how core methodologic elements were implemented. Our study highlights the need to improve clarity, transparency, and methodologic rigor in both the design and execution of TTE. By proposing a structured framework for target trial specification and outlining practical considerations for implementation, we aimed to complement existing reporting standards and contribute to more robust, credible, and reproducible TTE research.

## Notes

Supplement 1. **eAppendix 1.** Search Strategy **eAppendix 2.** Automated Screening Using the fPIS-ML Technique **eAppendix 3.** Data Abstraction Form **eAppendix 4.** Expert Consultation Questionnaire **eFigure 1.** Flow Chart of Study Selection **eFigure 2.** The Trend of TTE Studies From 2017 to 2023 and the Distribution of Corresponding Author Country **eFigure 3.** The Causal Contrast Declared in the Target Trials vs in the Emulated Trials **eTable 1.** General Characteristics of Included TTE Studies **eTable 2.** The Methodological Characteristics for Implementing a TTE Study in the Included TTE Studies **eAppendix 5.** A Structured Framework for Designing Target Trials

Supplement 2.
Data Sharing Statement
