# Supplementary material for: Design and Implementation of Observational Studies Emulating a Target Trial
Source: JAMA Netw Open. 2026 Feb 19;9(2):e2558262. doi: 10.1001/jamanetworkopen.2025.58262 (PMC12921535; doi:10.1001/jamanetworkopen.2025.58262)
Supplement: Supplement 1. — eAppendix 1. Search Strategy eAppendix 2. Automated Screening Using the fPIS-ML Technique eAppendix 3. Data Abstraction Form eAppendix 4. Expert Consultation Questionnaire eFigure 1. Flow Chart of Study Selection eFigure 2. The Trend of TTE Studies From 2017 to 2023 and the Distribution of Corresponding Author Country eFigure 3. The Causal Contrast Declared in the Target Trials vs in the Emulated Trials eTable 1. General Characteristics of Included TTE Studies eTable 2. The Methodological Characteristics for Implementing a TTE Study in the Included TTE Studies eAppendix 5. A Structured Framework for Designing Target Trials [file jamanetwopen-e2558262-s001.pdf]

Supplementary Online Content

Ren Y, Jia Y, Liu L, et al. Design and implementation of observational studies emulating a target trial. *JAMA Netw Open*. 2026;9(2):e2558262.
doi:10.1001/jamanetworkopen.2025.58262

eAppendix 1. Search Strategy
eAppendix 2. Automated Screening Using the fPIS-ML Technique
eAppendix 3. Data Abstraction Form
eAppendix 4. Expert Consultation Questionnaire
eFigure 1. Flow Chart of Study Selection
eFigure 2. The Trend of TTE Studies From 2017 to 2023 and the Distribution of Corresponding Author Country
eFigure 3. The Causal Contrast Declared in the Target Trials vs in the Emulated Trials
eTable 1. General Characteristics of Included TTE Studies
eTable 2. The Methodological Characteristics for Implementing a TTE Study in the Included TTE Studies
eAppendix 5. A Structured Framework for Designing Target Trials

This supplementary material has been provided by the authors to give readers additional information about their work.

© 2026 Ren Y et al. *JAMA Network Open*.

eAppendix 1. Search strategy

PubMed (Search on December 15, 2023)

1. ((((("retrospective database"[tiab] OR "secondary data"[tiab] OR "medical insurance" [tiab] OR "datalink"[tiab])) OR ((Administrative[tiab] OR Claims[tiab] OR "routine data" [tiab] OR "routinely collected" [tiab]))) OR (("Databases as Topic"[mh] OR database*[tiab] OR "health care databases"[tiab] OR "healthcare databases"[tiab] OR "health care database"[tiab] OR "healthcare database"[tiab] OR "healthcare data"[tiab] OR "health care data"[tiab] OR "national database"[tiab]))) OR (((((((((((observational*) OR (big data)) OR (case control study)) OR (cohort analysis)) OR (cohort study)) OR (electronic medical record*)) OR (electronic health record*)) OR (routine* collect* data)) OR (real-world)) OR (data base)) OR (healthcare database*)) OR (health care))) AND ((((((target trial*) OR (target trial emulat*)) OR ((trial*) AND ((emulat*) OR (mimic)))) OR (non-randomised trial*)) OR (hypothetic trial*)) OR (((comparative safety) OR (comparative effectiveness)) AND (trial*))))
2. ((trial* emulat*[Title/Abstract]) OR (emulat* trial*[Title/Abstract])) OR (target* trial*[Title/Abstract])
3. #1 OR #2
4. "2017/01/01"[Date - Publication] : "2023/12/15"[Date - Publication]
5. #3 AND #4
6. (”SEMIN NUCL MED“[Journal]) OR (”ACTA DERM-VENEREOL“[Journal]) OR (”ACTA NEUROPATHOL“[Journal]) OR (”AGE AGEING“[Journal]) OR (”J AM DENT ASSOC“[Journal]) OR (”AM FAM PHYSICIAN“[Journal]) OR (”J AM GERIATR SOC“[Journal]) OR (”AM J CLIN NUTR“[Journal]) OR (”AM J EPIDEMIOL“[Journal]) OR (”AM J GASTROENTEROL“[Journal]) OR (”AM J HUM GENET“[Journal]) OR (”AM J MED“[Journal]) OR (”AM J NURS“[Journal]) OR (”AM J OBSTET GYNECOL“[Journal]) OR (”AM J OPHTHALMOL“[Journal]) OR (”AM J PATHOL“[Journal]) OR (”AM J TROP MED HYG“[Journal]) OR (”ANAESTHESIA“[Journal]) OR (”ANESTH ANALG“[Journal]) OR (”ANESTHESIOLOGY“[Journal]) OR (”ANN INTERN MED“[Journal]) OR (”ANN SURG“[Journal]) OR (”ANN RHEUM DIS“[Journal]) OR (”ANN THORAC SURG“[Journal]) OR (”ARCH DIS CHILD“[Journal]) OR (”ARCH PATHOL LAB MED“[Journal]) OR (”BEHAV RES THER“[Journal]) OR (”BIOCHEM PHARMACOL“[Journal]) OR (”BLOOD“[Journal]) OR (”BRAIN“[Journal]) OR (”CA-CANCER J CLIN“[Journal]) OR (”CAN J OPHTHALMOL“[Journal]) OR (”CANCER RES“[Journal]) OR (”CARDIOVASC RES“[Journal]) OR (”CARIES RES“[Journal]) OR (”CHEM-BIOL INTERACT“[Journal]) OR (”CIRCULATION“[Journal]) OR (”CIRC RES“[Journal]) OR (”CLIN CHIM ACTA“[Journal]) OR (”CLIN CHEM“[Journal]) OR (”CLIN PHARMACOL THER“[Journal]) OR (”COMPUT BIOL MED“[Journal]) OR (”CORTEX“[Journal]) OR (”INT J DERMATOL“[Journal]) OR (”DEV MED CHILD NEUROL“[Journal]) OR (”DIABETES“[Journal]) OR (”DIABETOLOGIA“[Journal]) OR (”CHEST“[Journal]) OR (”DIS COLON RECTUM“[Journal]) OR (”DRUGS“[Journal]) OR (”ENDOSCOPY“[Journal]) OR (”ENVIRON RES“[Journal]) OR (”EPILEPSIA“[Journal]) OR (”EUR J CLIN INVEST“[Journal]) OR (”EUR J PHARMACOL“[Journal]) OR (”EXP NEUROL“[Journal]) OR (”FERTIL STERIL“[Journal]) OR (”GASTROENTEROLOGY“[Journal]) OR (”GASTROINTEST ENDOSC“[Journal]) OR (”GUT“[Journal]) OR (”HEADACHE“[Journal]) OR (”HORM BEHAV“[Journal]) OR (”INDIAN J PEDIATR“[Journal]) OR (”INT J CANCER“[Journal]) OR (”INT J NURS STUD“[Journal]) OR (”INT NURS REV“[Journal]) OR (”INVEST RADIOL“[Journal]) OR (”J ABNORM PSYCHOL“[Journal]) OR (”J BONE JOINT SURG AM“[Journal]) OR (”J CLIN INVEST“[Journal]) OR (”J DENT RES“[Journal]) OR (”J EXP MED“[Journal]) OR (”J HEALTH SOC BEHAV“[Journal]) OR (”J INFECT DIS“[Journal]) OR (”J INVEST DERMATOL“[Journal]) OR (”J NEUROSURG“[Journal]) OR (”J PATHOL“[Journal]) OR (”J PERIODONTOL“[Journal]) OR (”J PROSTHET DENT“[Journal]) OR (”LAB INVEST“[Journal]) OR (”LIFE SCI“[Journal]) OR (”MAYO CLIN PROC“[Journal]) OR (”METABOLISM“[Journal]) OR (”NEUROLOGY“[Journal]) OR (”NEUROPHARMACOLOGY“[Journal]) OR (”NURS OUTLOOK“[Journal]) OR (”NUTR REV“[Journal]) OR (”OBSTET GYNECOL SURV“[Journal]) OR (”OBSTET GYNECOL“[Journal]) OR (”PATHOLOGY“[Journal]) OR (”PEDIATR RES“[Journal]) OR (”PEDIATRICS“[Journal]) OR (”PHARMACOL REV“[Journal]) OR (”PHYS THER“[Journal]) OR (”PHYSIOTHERAPY“[Journal]) OR (”PLAST RECONSTR SURG“[Journal]) OR (”POSTGRAD MED J“[Journal]) OR (”PROG CARDIOVASC DIS“[Journal]) OR (”PSYCHOL MED“[Journal]) OR (”PSYCHOTHER PSYCHOSOM“[Journal]) OR (”PUBLIC HEALTH“[Journal]) OR (”RADIOL MED“[Journal]) OR (”RADIOLOGY“[Journal]) OR (”STROKE“[Journal]) OR (”SURGERY“[Journal]) OR (”SURV OPHTHALMOL“[Journal]) OR (”THEOR APPL GENET“[Journal]) OR (”THORAX“[Journal]) OR

© 2026 Ren Y et al. *JAMA Network Open*.

("TRANSPLANTATION"[Journal]) OR ("THROMB RES"[Journal]) OR ("ANNU REV GENET"[Journal]) OR ("ANNU REV MED"[Journal]) OR ("KIDNEY INT"[Journal]) OR ("AM J PUBLIC HEALTH"[Journal]) OR ("CRIT CARE MED"[Journal]) OR ("REHABIL PSYCHOL"[Journal]) OR ("GYNECOL ONCOL"[Journal]) OR ("EXERC SPORT SCI REV"[Journal]) OR ("PREV MED"[Journal]) OR ("SEMIN THROMB HEMOST"[Journal]) OR ("MOL ASPECTS MED"[Journal]) OR ("CONTACT DERMATITIS"[Journal]) OR ("IMMUNOL REV"[Journal]) OR ("ALLERGY"[Journal]) OR ("DENT MATER"[Journal]) OR ("SPORTS MED"[Journal]) OR ("DRUG SAFETY"[Journal]) OR ("BEHAV BRAIN SCI"[Journal]) OR ("LANCET"[Journal]) OR ("J INHERIT METAB DIS"[Journal]) OR ("MED TEACH"[Journal]) OR ("BIOMATERIALS"[Journal]) OR ("INT ENDOD J"[Journal]) OR ("CLIN SCI"[Journal]) OR ("SEMIN PERINATOL"[Journal]) OR ("J MED VIROL"[Journal]) OR ("ANNU REV NEUROSCI"[Journal]) OR ("AM J SURG PATHOL"[Journal]) OR ("HEART LUNG"[Journal]) OR ("NEUROSURGERY"[Journal]) OR ("J MED SYST"[Journal]) OR ("DIABETES CARE"[Journal]) OR ("J NUCL MED"[Journal]) OR ("OPHTHALMOLOGY"[Journal]) OR ("SLEEP"[Journal]) OR ("CANCER NURS"[Journal]) OR ("J NAT PROD"[Journal]) OR ("ENDOCR REV"[Journal]) OR ("PROG LIPID RES"[Journal]) OR ("TRENDS PHARMACOL SCI"[Journal]) OR ("TRENDS NEUROSCI"[Journal]) OR ("AQUAT TOXICOL"[Journal]) OR ("RADIOTHER ONCOL"[Journal]) OR ("J CONTROL RELEASE"[Journal]) OR ("J HEPATOL"[Journal]) OR ("TRENDS GENET"[Journal]) OR ("TRENDS ECOL EVOL"[Journal]) OR ("ARCH MED RES"[Journal]) OR ("J AM ACAD DERMATOL"[Journal]) OR ("EPIDEMIOL REV"[Journal]) OR ("HYPERTENSION"[Journal]) OR ("APPETITE"[Journal]) OR ("EUR HEART J"[Journal]) OR ("J HOSP INFECT"[Journal]) OR ("ANN EMERG MED"[Journal]) OR ("AM J INFECT CONTROL"[Journal]) OR ("GERIATR NURS"[Journal]) OR ("MED RES REV"[Journal]) OR ("ANNU REV NUTR"[Journal]) OR ("BIOCYBERN BIOMED ENG"[Journal]) OR ("AM J NEPHROL"[Journal]) OR ("NEUROEPIDEMIOLOGY"[Journal]) OR ("ARCH PHARM RES"[Journal]) OR ("CLIN NUTR"[Journal]) OR ("MIDWIFERY"[Journal]) OR ("HUM REPROD"[Journal]) OR ("BLOOD REV"[Journal]) OR ("CLIN REHABIL"[Journal]) OR ("J NEUROSCI"[Journal]) OR ("HEPATOLOGY"[Journal]) OR ("RADIOGRAPHICS"[Journal]) OR ("J CLIN IMMUNOL"[Journal]) OR ("AM J KIDNEY DIS"[Journal]) OR ("CLIN PSYCHOL REV"[Journal]) OR ("AM J OCCUP THER"[Journal]) OR ("SOC SCI MED"[Journal]) OR ("HEALTH PSYCHOL"[Journal]) OR ("FOOD CHEM TOXICOL"[Journal]) OR ("RHINOLOGY"[Journal]) OR ("ARCH BRONCONEUMOL"[Journal]) OR ("TOXICOLOGY"[Journal]) OR ("J DENT"[Journal]) OR ("INT J EPIDEMIOL"[Journal]) OR ("INFECTION"[Journal]) OR ("BASIC RES CARDIOL"[Journal]) OR ("RESUSCITATION"[Journal]) OR ("PROG NEUROBIOL"[Journal]) OR ("EUR UROL"[Journal]) OR ("J CLIN PERIODONTOL"[Journal]) OR ("CANCER LETT"[Journal]) OR ("PAIN"[Journal]) OR ("ANN ACAD MED SINGAP"[Journal]) OR ("CANCER TREAT REV"[Journal]) OR ("ADDICT BEHAV"[Journal]) OR ("J MED ETHICS"[Journal]) OR ("FOOD POLICY"[Journal]) OR ("CLIN EXP DERMATOL"[Journal]) OR ("MED EDUC"[Journal]) OR ("FOOD CHEM"[Journal]) OR ("HISTOPATHOLOGY"[Journal]) OR ("J ADV NURS"[Journal]) OR ("CEPHALALGIA"[Journal]) OR ("J NEUROL"[Journal]) OR ("ARCH TOXICOL"[Journal]) OR ("EUR J PEDIATR"[Journal]) OR ("HUM GENET"[Journal]) OR ("DRUG METAB REV"[Journal]) OR ("AM J HEMATOL"[Journal]) OR ("ANNU REV PHARMACOL"[Journal]) OR ("CLIN NUCL MED"[Journal]) OR ("ANN NEUROL"[Journal]) OR ("MATURITAS"[Journal]) OR ("J ETHNOPHARMACOL"[Journal]) OR ("FORENSIC SCI INT"[Journal]) OR ("CHEM SENSES"[Journal]) OR ("HAEMATOLOGICA"[Journal]) OR ("J ENDOCRINOL INVEST"[Journal]) OR ("EUR J EPIDEMIOL"[Journal]) OR ("ANNU REV IMMUNOL"[Journal]) OR ("J CLIN ONCOL"[Journal]) OR ("J AM COLL CARDIOL"[Journal]) OR ("AM J EMERG MED"[Journal]) OR ("MOL BIOL EVOL"[Journal]) OR ("J VASC SURG"[Journal]) OR ("CELL BIOL TOXICOL"[Journal]) OR ("J PINEAL RES"[Journal]) OR ("AM J PREV MED"[Journal]) OR ("ARTHROSCOPY"[Journal]) OR ("BIOMED PHARMACOTHER"[Journal]) OR ("ACTA PAEDIATR"[Journal]) OR ("EUR J ENDOCRINOL"[Journal]) OR ("CAN MED ASSOC J"[Journal]) OR ("CAN J CARDIOL"[Journal]) OR ("HEALTH REP"[Journal]) OR ("BIOL SPORT"[Journal]) OR ("J ARTHROPLASTY"[Journal]) OR ("J BONE MINER RES"[Journal]) OR ("J GEN INTERN MED"[Journal]) OR ("MILBANK Q"[Journal]) OR ("J ANXIETY DISORD"[Journal]) OR ("LEUKEMIA"[Journal]) OR ("PEDIATR NEUROL"[Journal]) OR ("GENOMICS"[Journal]) OR ("BRAIN BEHAV IMMUN"[Journal]) OR ("J HUM LACT"[Journal]) OR ("J RURAL HEALTH"[Journal]) OR ("PEDIATR INFECT DIS J"[Journal]) OR ("RES DEV DISABIL"[Journal]) OR ("GLIA"[Journal]) OR ("J CLIN EPIDEMIOL"[Journal]) OR ("NEURON"[Journal]) OR ("J AUTOIMMUN"[Journal]) OR ("J DIGIT IMAGING"[Journal]) OR ("ACCOUNT RES"[Journal]) OR ("EUR RESPIR J"[Journal]) OR ("INDOOR AIR"[Journal]) OR ("EUR RESPIR REV"[Journal]) OR ("EXP DERMATOL"[Journal]) OR ("PERIODONTOL

© 2026 Ren Y et al. *JAMA Network Open*.

2000“[Journal]) OR (”HYPERTENS RES“[Journal]) OR (”J DERMATOL SCI“[Journal]) OR (”ANN ONCOL“[Journal]) OR (”INFLAMMOPHARMACOLOGY“[Journal]) OR (”CANCER GENE THER“[Journal]) OR (”SURG ENDOSC“[Journal]) OR (”ARTIF INTELL MED“[Journal]) OR (”MYCOSES“[Journal]) OR (”TRANSPL INT“[Journal]) OR (”EUR RADIOL“[Journal]) OR (”EUR J PHARM BIOPHARM“[Journal]) OR (”SUPPORT CARE CANCER“[Journal]) OR (”J GASTROENTEROL“[Journal]) OR (”PHYTOMEDICINE“[Journal]) OR (”J MOL MED“[Journal]) OR (”ONCOGENE“[Journal]) OR (”PHYTOTHER RES“[Journal]) OR (”BIOFACTORS“[Journal]) OR (”CURR OPIN IMMUNOL“[Journal]) OR (”J CLIN ANESTH“[Journal]) OR (”EUR J INTERN MED“[Journal]) OR (”NUTR RES REV“[Journal]) OR (”J INTERN MED“[Journal]) OR (”CLIN EXP ALLERGY“[Journal]) OR (”J NUTR BIOCHEM“[Journal]) OR (”INT J DRUG POLICY“[Journal]) OR (”CURR OPIN NEUROBIOL“[Journal]) OR (”EUR J CANCER“[Journal]) OR (”CLIN AUTON RES“[Journal]) OR (”INT J PAEDIATR DENT“[Journal]) OR (”BREAST“[Journal]) OR (”J CLIN NURS“[Journal]) OR (”TOB CONTROL“[Journal]) OR (”ADDICTION“[Journal]) OR (”DISABIL SOC“[Journal]) OR (”ANGIOGENESIS“[Journal]) OR (”GENE THER“[Journal]) OR (”NURS ETHICS“[Journal]) OR (”EUR J EMERG MED“[Journal]) OR (”NEUROBIOL DIS“[Journal]) OR (”PATHOBIOLOGY“[Journal]) OR (”BRAIN PATHOL“[Journal]) OR (”ASIAN J SURG“[Journal]) OR (”EUR J HUM GENET“[Journal]) OR (”J BIOMED SCI“[Journal]) OR (”AUST CRIT CARE“[Journal]) OR (”ACAD MED“[Journal]) OR (”NEUROPSYCHOL REV“[Journal]) OR (”CRIT REV TOXICOL“[Journal]) OR (”CURR OPIN PEDIATR“[Journal]) OR (”CURR OPIN OPHTHALMOL“[Journal]) OR (”HEALTH COMMUN“[Journal]) OR (”INT PSYCHOGERIATR“[Journal]) OR (”HUM GENE THER“[Journal]) OR (”PHARMACOL RES“[Journal]) OR (”EPIDEMIOLOGY“[Journal]) OR (”SEMIN IMMUNOL“[Journal]) OR (”SEMIN CANCER BIOL“[Journal]) OR (”ENDOCR PATHOL“[Journal]) OR (”J AM SOC NEPHROL“[Journal]) OR (”ANN EPIDEMIOL“[Journal]) OR (”INT J GYNECOL CANCER“[Journal]) OR (”THYROID“[Journal]) OR (”REV MED VIROL“[Journal]) OR (”J OCCUP REHABIL“[Journal]) OR (”J MAGN RESON IMAGING“[Journal]) OR (”NEUROIMAGE“[Journal]) OR (”MMWR RECOMM REP“[Journal]) OR (”CLIN INFECT DIS“[Journal]) OR (”J PROSTHODONT“[Journal]) OR (”NAT GENET“[Journal]) OR (”AM J CRIT CARE“[Journal]) OR (”HUM BRAIN MAPP“[Journal]) OR (”ANN SURG ONCOL“[Journal]) OR (”ACAD EMERG MED“[Journal]) OR (”DRUG DELIV“[Journal]) OR (”J CARD FAIL“[Journal]) OR (”ADV ANAT PATHOL“[Journal]) OR (”EUR EAT DISORD REV“[Journal]) OR (”SHOCK“[Journal]) OR (”NEUROSCIENTIST“[Journal]) OR (”SEMIN ORTHOD“[Journal]) OR (”IMMUNITY“[Journal]) OR (”J FAM NURS“[Journal]) OR (”ACAD RADIOL“[Journal]) OR (”EXERC IMMUNOL REV“[Journal]) OR (”CLIN CANCER RES“[Journal]) OR (”NAT MED“[Journal]) OR (”EMERG INFECT DIS“[Journal]) OR (”ONCOLOGIST“[Journal]) OR (”SLEEP MED REV“[Journal]) OR (”GENOME RES“[Journal]) OR (”EVOL HUM BEHAV“[Journal]) OR (”AESTHET SURG J“[Journal]) OR (”J GASTROINTEST SURG“[Journal]) OR (”DEPRESS ANXIETY“[Journal]) OR (”NEUROSURG FOCUS“[Journal]) OR (”PHYSIOL GENOMICS“[Journal]) OR (”NAT NEUROSCI“[Journal]) OR (”VALUE HEALTH“[Journal]) OR (”GENET MED“[Journal]) OR (”J URBAN HEALTH“[Journal]) OR (”EUROPACE“[Journal]) OR (”TECH COLOPROCTOL“[Journal]) OR (”J HEADACHE PAIN“[Journal]) OR (”EMERGENCIAS“[Journal]) OR (”PHARMACOECONOMICS“[Journal]) OR (”CNS DRUGS“[Journal]) OR (”BIODRUGS“[Journal]) OR (”AM J CLIN DERMATOL“[Journal]) OR (”J TRAVEL MED“[Journal]) OR (”INT J INFECT DIS“[Journal]) OR (”EXP MOL MED“[Journal]) OR (”BIOMATER RES“[Journal]) OR (”J GINSENG RES“[Journal]) OR (”KOREAN J RADIOL“[Journal]) OR (”DIABETES METAB“[Journal]) OR (”J NUTR HEALTH AGING“[Journal]) OR (”MICROBES INFECT“[Journal]) OR (”RESPIROLOGY“[Journal]) OR (”ALLERGOL INT“[Journal]) OR (”AMYLOID“[Journal]) OR (”CURR OPIN NEUROL“[Journal]) OR (”PROG RETIN EYE RES“[Journal]) OR (”OCCUP ENVIRON MED“[Journal]) OR (”EUR J NEUROL“[Journal]) OR (”HEALTH PLACE“[Journal]) OR (”EXPERT OPIN THER PAT“[Journal]) OR (”ORAL DIS“[Journal]) OR (”HUM REPROD UPDATE“[Journal]) OR (”J TELEMED TELECARE“[Journal]) OR (”DRUG DISCOV TODAY“[Journal]) OR (”TROP MED INT HEALTH“[Journal]) OR (”MOL HUM REPROD“[Journal]) OR (”MED IMAGE ANAL“[Journal]) OR (”NURS CRIT CARE“[Journal]) OR (”TRENDS COGN SCI“[Journal]) OR (”CRIT CARE“[Journal]) OR (”ORAL ONCOL“[Journal]) OR (”EUR J GEN PRACT“[Journal]) OR (”INT J MED INFORM“[Journal]) OR (”J CLIN VIROL“[Journal]) OR (”CLIN NEUROPHYSIOL“[Journal]) OR (”EUR J HEART FAIL“[Journal]) OR (”SLEEP MED“[Journal]) OR (”DERMATOL THER“[Journal]) OR (”BIPOLAR DISORD“[Journal]) OR (”PEDIATR DIABETES“[Journal]) OR (”BRAZ J PHYS THER“[Journal]) OR (”CLIN CHEM LAB MED“[Journal]) OR (”GASTRIC CANCER“[Journal]) OR (”J MED INTERNET RES“[Journal]) OR (”J SCI MED SPORT“[Journal]) OR (”NURS HEALTH SCI“[Journal])

© 2026 Ren Y et al. *JAMA Network Open*.

OR ("CLIN EXP OPHTHALMOL"[Journal]) OR ("RHEUMATOLOGY"[Journal]) OR ("NICOTINE TOB RES"[Journal]) OR ("EUR J ONCOL NURS"[Journal]) OR ("EXPERT REV MOL MED"[Journal]) OR ("DIABETES OBES METAB"[Journal]) OR ("COLORECTAL DIS"[Journal]) OR ("BJU INT"[Journal]) OR ("BREAST CANCER RES"[Journal]) OR ("GENES IMMUN"[Journal]) OR ("OBES REV"[Journal]) OR ("PSYCHOL SPORT EXERC"[Journal]) OR ("LANCET ONCOL"[Journal]) OR ("NAT REV NEUROSCI"[Journal]) OR ("NAT REV GENET"[Journal]) OR ("BMC GENOMICS"[Journal]) OR ("TRENDS IMMUNOL"[Journal]) OR ("TRENDS MOL MED"[Journal]) OR ("NURSE EDUC PRACT"[Journal]) OR ("REPROD BIOMED ONLINE"[Journal]) OR ("BMC NURS"[Journal]) OR ("LANCET INFECT DIS"[Journal]) OR ("EXPERT REV MOL DIAGN"[Journal]) OR ("HUM GENOMICS"[Journal]) OR ("NAT REV IMMUNOL"[Journal]) OR ("NAT REV CANCER"[Journal]) OR ("NAT REV DRUG DISCOV"[Journal]) OR ("LANCET NEUROL"[Journal]) OR ("GENOME BIOL"[Journal]) OR ("AGING CELL"[Journal]) OR ("INT J HEALTH GEOGR"[Journal]) OR ("LIVER INT"[Journal]) OR ("INT J AUDIOL"[Journal]) OR ("ENVIRON TOXICOL"[Journal]) OR ("CLIN IMMUNOL"[Journal]) OR ("CURR HYPERTENS REP"[Journal]) OR ("NEURO-ONCOLOGY"[Journal]) OR ("CURR ATHEROSCLER REP"[Journal]) OR ("ANNU REV BIOMED ENG"[Journal]) OR ("MOL THER"[Journal]) OR ("J AM MED DIR ASSOC"[Journal]) OR ("PAEDIATR RESPIR REV"[Journal]) OR ("NAT IMMUNOL"[Journal]) OR ("SPINE J"[Journal]) OR ("CANCER CELL"[Journal]) OR ("EPILEPSY CURR"[Journal]) OR ("J THROMB HAEMOST"[Journal]) OR ("OCUL SURF"[Journal]) OR ("ANN FAM MED"[Journal]) OR ("PREV CHRONIC DIS"[Journal]) OR ("MMWR SURVEILL SUMM"[Journal]) OR ("J AM COLL RADIOL"[Journal]) OR ("ANN AM THORAC SOC"[Journal]) OR ("PLOS MED"[Journal]) OR ("CELL METAB"[Journal]) OR ("SURG OBES RELAT DIS"[Journal]) OR ("ALZHEIMERS DEMENT"[Journal]) OR ("PLOS PATHOG"[Journal]) OR ("PLOS GENET"[Journal]) OR ("J THORAC ONCOL"[Journal]) OR ("J NEUROL PHYS THER"[Journal]) OR ("INT IMMUNOPHARMACOL"[Journal]) OR ("MITOCHONDRION"[Journal]) OR ("AGEING RES REV"[Journal]) OR ("AUTOIMMUN REV"[Journal]) OR ("CURR NEUROPHARMACOL"[Journal]) OR ("MOL ONCOL"[Journal]) OR ("EUR J PAEDIATR DENT"[Journal]) OR ("AM J TRANSPLANT"[Journal]) OR ("J DTSCH DERMATOL GES"[Journal]) OR ("EUR J HEALTH ECON"[Journal]) OR ("J REHABIL MED"[Journal]) OR ("INT J PUBLIC HEALTH"[Journal]) OR ("FRONT IMMUNOL"[Journal]) OR ("CARDIORENAL MED"[Journal]) OR ("ACTA PHARMACOL SIN"[Journal]) OR ("CELL MOL IMMUNOL"[Journal]) OR ("NEURAL REGEN RES"[Journal]) OR ("NEUROSCI BULL"[Journal]) OR ("J GENET GENOMICS"[Journal]) OR ("INT J ORAL SCI"[Journal]) OR ("INT BRAZ J UROL"[Journal]) OR ("J NURS RES"[Journal]) OR ("WORLD J PEDIATR"[Journal]) OR ("DERMATITIS"[Journal]) OR ("ITAL J PEDIATR"[Journal]) OR ("WORLD PSYCHIATRY"[Journal]) OR ("BODY IMAGE"[Journal]) OR ("CANCER IMAGING"[Journal]) OR ("MATERN CHILD NUTR"[Journal]) OR ("BMC MED"[Journal]) OR ("INT WOUND J"[Journal]) OR ("IMMUN AGEING"[Journal]) OR ("ACTA BIOMATER"[Journal]) OR ("NANOTOXICOLOGY"[Journal]) OR ("HEALTH PSYCHOL REV"[Journal]) OR ("PART FIBRE TOXICOL"[Journal]) OR ("INT J SURG"[Journal]) OR ("ACTA ORTHOP"[Journal]) OR ("INT BREASTFEED J"[Journal]) OR ("INT J STROKE"[Journal]) OR ("IMPLEMENT SCI"[Journal]) OR ("WORLD J EMERG SURG"[Journal]) OR ("CNS NEUROSCI THER"[Journal]) OR ("J FUNCT FOODS"[Journal]) OR ("J EVID-BASED MED"[Journal]) OR ("GENOME MED"[Journal]) OR ("EMBO MOL MED"[Journal]) OR ("BIOFABRICATION"[Journal]) OR ("NAT REV NEUROL"[Journal]) OR ("NAT REV CLIN ONCOL"[Journal]) OR ("NAT REV RHEUMATOL"[Journal]) OR ("NAT REV UROL"[Journal]) OR ("NAT REV CARDIOL"[Journal]) OR ("NAT REV ENDOCRINOL"[Journal]) OR ("NAT REV NEPHROL"[Journal]) OR ("J NEUROINTERV SURG"[Journal]) OR ("EUROINTERVENTION"[Journal]) OR ("ASIAN J PHARM SCI"[Journal]) OR ("J PHYSIOTHER"[Journal]) OR ("THERANOSTICS"[Journal]) OR ("SEMIN IMMUNOPATHOL"[Journal]) OR ("DTSCH ARZTEBL INT"[Journal]) OR ("J NEURODEV DISORD"[Journal]) OR ("TRANSL STROKE RES"[Journal]) OR ("CLIN EPIGENETICS"[Journal]) OR ("INSIGHTS IMAGING"[Journal]) OR ("WOMEN BIRTH"[Journal]) OR ("J CROHNS COLITIS"[Journal]) OR ("CLIN SIMUL NURS"[Journal]) OR ("ASIAN J PSYCHIATR"[Journal]) OR ("ANN PHYS REHABIL MED"[Journal]) OR ("EPMA J"[Journal]) OR ("CURR OPIN VIROL"[Journal]) OR ("JPN DENT SCI REV"[Journal]) OR ("J PROSTHODONT RES"[Journal]) OR ("REV ESP CARDIOL"[Journal]) OR ("CELL HOST MICROBE"[Journal]) OR ("TRANSL RES"[Journal]) OR ("J ADDICT MED"[Journal]) OR ("MUCOSAL IMMUNOL"[Journal]) OR ("NEUROTHERAPEUTICS"[Journal]) OR ("BRAIN STIMUL"[Journal]) OR ("HEPATOL INT"[Journal]) OR ("DISABIL HEALTH J"[Journal]) OR ("IEEE REV BIOMED ENG"[Journal]) OR ("AUTISM RES"[Journal]) OR ("CIRC-HEART FAIL"[Journal]) OR ("SCI TRANSL MED"[Journal]) OR ("GUT MICROBES"[Journal]) OR ("CLIN TRANSL MED"[Journal]) OR ("J

© 2026 Ren Y et al. *JAMA Network Open*.

GYNECOL ONCOL“[Journal]) OR (”MOL AUTISM“[Journal]) OR (”MOL ORAL MICROBIOL“[Journal]) OR (”NUTR DIABETES“[Journal]) OR (”BLOOD CANCER J“[Journal]) OR (”BMJ QUAL SAF“[Journal]) OR (”FLUIDS BARRIERS CNS“[Journal]) OR (”BONE JOINT RES“[Journal]) OR (”J GLOB HEALTH“[Journal]) OR (”EUR J PREV CARDIOL“[Journal]) OR (”PEDIATR OBES“[Journal]) OR (”PATHOG GLOB HEALTH“[Journal]) OR (”TRANSL NEURODEGENER“[Journal]) OR (”CLIN KIDNEY J“[Journal]) OR (”BONE JOINT J“[Journal]) OR (”J EAT DISORD“[Journal]) OR (”J MATER CHEM B“[Journal]) OR (”J INTENSIVE CARE“[Journal]) OR (”J LAW BIOSCI“[Journal]) OR (”DIGIT HEALTH“[Journal]) OR (”REGEN BIOMATER“[Journal]) OR (”BJPSYCH OPEN“[Journal]) OR (”RMD OPEN“[Journal]) OR (”NAT REV DIS PRIMERS“[Journal]) OR (”HLA“[Journal]) OR (”BMJ GLOB HEALTH“[Journal]) OR (”STROKE VASC NEUROL“[Journal]) OR (”J BEHAV ADDICT“[Journal]) OR (”ANTIBIOTICS-BASEL“[Journal]) OR (”J PHARM ANAL“[Journal]) OR (”J SPORT HEALTH SCI“[Journal]) OR (”INFECT DIS POVERTY“[Journal]) OR (”ANN INTENSIVE CARE“[Journal]) OR (”AGING DIS“[Journal]) OR (”KIDNEY INT SUPPL“[Journal]) OR (”NAT BIOMED ENG“[Journal]) OR (”ONCOGENESIS“[Journal]) OR (”QUAL RES SPORT EXERC“[Journal]) OR (”CANCER DISCOV“[Journal]) OR (”GAMES HEALTH J“[Journal]) OR (”ADV NUTR“[Journal]) OR (”ADV WOUND CARE“[Journal]) OR (”ONCOIMMUNOLOGY“[Journal]) OR (”CURR OBES REP“[Journal]) OR (”WORKPLACE HEALTH SAF“[Journal]) OR (”CLIN PSYCHOL SCI“[Journal]) OR (”JAMA DERMATOL“[Journal]) OR (”JAMA INTERN MED“[Journal]) OR (”JAMA NEUROL“[Journal]) OR (”JAMA OPHTHALMOL“[Journal]) OR (”JAMA PEDIATR“[Journal]) OR (”JAMA SURG“[Journal]) OR (”ADV HEALTHC MATER“[Journal]) OR (”PROG ORTHOD“[Journal]) OR (”CURR POLLUT REP“[Journal]) OR (”SPORTS MED-OPEN“[Journal]) OR (”CELL ONCOL“[Journal]) OR (”ACTA PHARM SIN B“[Journal]) OR (”MOL METAB“[Journal]) OR (”JACC-HEART FAIL“[Journal]) OR (”PHOTOACOUSTICS“[Journal]) OR (”LANCET GLOB HEALTH“[Journal]) OR (”DIABETES METAB J“[Journal]) OR (”ANN LAB MED“[Journal]) OR (”LIVER CANCER“[Journal]) OR (”CLIN MOL HEPATOL“[Journal]) OR (”WORLD J MENS HEALTH“[Journal]) OR (”J STROKE“[Journal]) OR (”JMIR MHEALTH UHEALTH“[Journal]) OR (”KIDNEY DIS-BASEL“[Journal]) OR (”BURNS TRAUMA“[Journal]) OR (”LGBT HEALTH“[Journal]) OR (”ARTHRITIS RHEUMATOL“[Journal]) OR (”CANCER IMMUNOL RES“[Journal]) OR (”ANNU REV VIROL“[Journal]) OR (”HEALTH SYST REFORM“[Journal]) OR (”NEUROPHOTONICS“[Journal]) OR (”TRENDS HEAR“[Journal]) OR (”CURR OPIN BEHAV SCI“[Journal]) OR (”LANCET HIV“[Journal]) OR (”LANCET HAEMATOL“[Journal]) OR (”EBIOMEDICINE“[Journal]) OR (”GENES DIS“[Journal]) OR (”JAMA ONCOL“[Journal]) OR (”ANNU REV VIS SCI“[Journal]) OR (”JAMA CARDIOL“[Journal]) OR (”EFORT OPEN REV“[Journal]) OR (”EUR STROKE J“[Journal]) OR (”NAT HUM BEHAV“[Journal]) OR (”NPJ DIGIT MED“[Journal]) OR (”TRENDS CANCER“[Journal]) OR (”KIDNEY INT REP“[Journal]) OR (”LANCET PUBLIC HEALTH“[Journal]) OR (”SCI IMMUNOL“[Journal]) OR (”ANNU REV CANCER BIOL“[Journal]) OR (”APL BIOENG“[Journal]) OR (”BLOOD ADV“[Journal]) OR (”BJS OPEN“[Journal]) OR (”BMJ EVID-BASED MED“[Journal]) OR (”PULMONOLOGY“[Journal]) OR (”JOR SPINE“[Journal]) OR (”JAMA NETW OPEN“[Journal]) OR (”NEUROSPINE“[Journal]) OR (”MATER TODAY BIO“[Journal]) OR (”PHYS ENG SCI MED“[Journal]) OR (”LANCET RHEUMATOL“[Journal]) OR (”JACC-CARDIOONCOL“[Journal]) OR (”CELL REP MED“[Journal]) OR (”MINERVA UROL NEPHROL“[Journal]) OR (”CARDIOVASC DIABETOL“[Journal]) OR (”CANCER CELL INT“[Journal]) OR (”INT J EQUITY HEALTH“[Journal]) OR (”MOL CANCER“[Journal]) OR (”HARM REDUCT J“[Journal]) OR (”WORLD J SURG ONCOL“[Journal]) OR (”HUM RESOUR HEALTH“[Journal]) OR (”J TRANSL MED“[Journal]) OR (”MAR DRUGS“[Journal]) OR (”FRONT CELL NEUROSCI“[Journal]) OR (”FRONT PHARMACOL“[Journal]) OR (”J NEUROENG REHABIL“[Journal]) OR (”BEHAV BRAIN FUNCT“[Journal]) OR (”MOL NEURODEGENER“[Journal]) OR (”J HEMATOL ONCOL“[Journal]) OR (”J OVARIAN RES“[Journal]) OR (”STEM CELL RES THER“[Journal]) OR (”J INT AIDS SOC“[Journal]) OR (”ALZHEIMERS RES THER“[Journal]) OR (”INFLAMM REGEN“[Journal]) OR (”PHARMACEUTICS“[Journal]) OR (”BIOL SEX DIFFER“[Journal]) OR (”CANCER METAB“[Journal]) OR (”BIOMARK RES“[Journal]) OR (”J IMMUNOTHER CANCER“[Journal]) OR (”NPJ GENOM MED“[Journal]) OR (”VIRUS EVOL“[Journal]) OR (”NPJ REGEN MED“[Journal]) OR (”NPJ VACCINES“[Journal]) OR (”ESMO OPEN“[Journal]) OR (”NUTRIENTS“[Journal]) OR (”TOXINS“[Journal]) OR (”VACCINES-BASEL“[Journal]) OR (”CHINA CDC WEEKLY“[Journal]) OR (”EXP HEMATOL ONCOL“[Journal]) OR (”CURR ADDICT REP“[Journal]) OR (”INTERNET INTERV“[Journal]) OR (”BIOMEDICINES“[Journal]) OR (”FRONT PUBLIC HEALTH“[Journal]) OR (”TOXICS“[Journal]) OR (”NPJ PARKINSONS DIS“[Journal]) OR (”NPJ BREAST CANCER“[Journal]) OR (”JCI INSIGHT“[Journal]) OR (”BIOENG TRANSL MED“[Journal]) OR

© 2026 Ren Y et al. *JAMA Network Open*.

(”NPJ PRECIS ONCOL“[Journal]) OR (”HUM REPROD OPEN“[Journal]) OR (”INNOV AGING“[Journal]) OR (”EUR UROL FOCUS“[Journal]) OR (”BIOACT MATER“[Journal]) OR (”NAT METAB“[Journal]) OR (”CANCER COMMUN“[Journal]) OR (”ENVIRON MICROBIOME“[Journal]) OR (”LANCET PLANET HEALTH“[Journal]) OR (”HEMASPHERE“[Journal]) OR (”EUR UROL ONCOL“[Journal]) OR (”ECLINICALMEDICINE“[Journal]) OR (”JHEP REP“[Journal]) OR (”LANCET DIGIT HEALTH“[Journal]) OR (”INT J PHARM-X“[Journal]) OR (”NAT CANCER“[Journal]) OR (”LANCET MICROBE“[Journal]) OR (”Accid Anal Prev“[Journal]) OR (”Acta Neuropathol Commun“[Journal]) OR (”Acta Obstet Gynecol Scand“[Journal]) OR (”Acta Psychiatr Scand“[Journal]) OR (”Adv Drug Deliv Rev“[Journal]) OR (”AIDS Patient Care STDS“[Journal]) OR (”AJR Am J Roentgenol“[Journal]) OR (”Alcohol Res Health“[Journal]) OR (”Aliment Pharmacol Ther“[Journal]) OR (”Am J Obstet Gynecol MFM“[Journal]) OR (”Am J Phys Med Rehabil“[Journal]) OR (”Am J Physiol Renal Physiol“[Journal]) OR (”Am J Respir Crit Care Med“[Journal]) OR (”Am J Respir Cell Mol Biol“[Journal]) OR (”Am J Speech Lang Pathol“[Journal]) OR (”Anaesth Crit Care Pain Med“[Journal]) OR (”Andrology“[Journal]) OR (”Ann Cardiothorac Surg“[Journal]) OR (”Ann Clin Transl Neurol“[Journal]) OR (”Annu Rev Clin Psychol“[Journal]) OR (”Annu Rev Genomics Hum Genet“[Journal]) OR (”Annu Rev Pathol“[Journal]) OR (”Annu Rev Public Health“[Journal]) OR (”Antimicrob Agents Chemother“[Journal]) OR (”Antimicrob Resist Infect Control“[Journal]) OR (”Antioxid Redox Signal“[Journal]) OR (”Antiviral Res“[Journal]) OR (”Appl Physiol Nutr Metab“[Journal]) OR (”Arch Pharm (Weinheim)“[Journal]) OR (”Arch Dis Child Fetal Neonatal Ed“[Journal]) OR (”Arch Phys Med Rehabil“[Journal]) OR (”Arterioscler Thromb Vasc Biol“[Journal]) OR (”Artif Cells Nanomed Biotechnol“[Journal]) OR (”Asia Pac J Ophthalmol (Phila)“[Journal]) OR (”Best Pract Res Clin Endocrinol Metab“[Journal]) OR (”Best Pract Res Clin Obstet Gynaecol“[Journal]) OR (”Best Pract Res Clin Rheumatol“[Journal]) OR (”Biochim Biophys Acta Rev Cancer“[Journal]) OR (”Biol Psychiatry“[Journal]) OR (”Biol Psychiatry Cogn Neurosci Neuroimaging“[Journal]) OR (”BJOG“[Journal]) OR (”BMJ“[Journal]) OR (”Bone Marrow Transplant“[Journal]) OR (”Breast Cancer“[Journal]) OR (”Br J Anaesth“[Journal]) OR (”Br J Cancer“[Journal]) OR (”Br J Haematol“[Journal]) OR (”Br J Health Psychol“[Journal]) OR (”Br J Pharmacol“[Journal]) OR (”Br J Sports Med“[Journal]) OR (”Br Med Bull“[Journal]) OR (”Bull World Health Organ“[Journal]) OR (”Cancer Immunol Immunother“[Journal]) OR (”Cancer Metastasis Rev“[Journal]) OR (”Cancer“[Journal]) OR (”Cell Mol Gastroenterol Hepatol“[Journal]) OR (”Child Adolesc Ment Health“[Journal]) OR (”Child Adolesc Psychiatry Ment Health“[Journal]) OR (”Chin J Nat Med“[Journal]) OR (”Chin Med J (Engl)“[Journal]) OR (”Chin Med“[Journal]) OR (”Circ Arrhythm Electrophysiol“[Journal]) OR (”Circ Cardiovasc Imaging“[Journal]) OR (”Circ Cardiovasc Qual Outcomes“[Journal]) OR (”Circ Genom Precis Med“[Journal]) OR (”Cleve Clin J Med“[Journal]) OR (”Clin Exp Otorhinolaryngol“[Journal]) OR (”Clin Child Fam Psychol Rev“[Journal]) OR (”Clin Gastroenterol Hepatol“[Journal]) OR (”Clin Implant Dent Relat Res“[Journal]) OR (”Clin J Am Soc Nephrol“[Journal]) OR (”Clin Microbiol Infect“[Journal]) OR (”Clin Oral Implants Res“[Journal]) OR (”Clin Orthop Relat Res“[Journal]) OR (”Clin Rev Allergy Immunol“[Journal]) OR (”Cogn Behav Ther“[Journal]) OR (”Compr Psychiatry“[Journal]) OR (”Comput Methods Programs Biomed“[Journal]) OR (”Comput Med Imaging Graph“[Journal]) OR (”Crit Rev Clin Lab Sci“[Journal]) OR (”Crit Rev Food Sci Nutr“[Journal]) OR (”Crit Rev Oncol Hematol“[Journal]) OR (”Curr Environ Health Rep“[Journal]) OR (”Curr Neurol Neurosci Rep“[Journal]) OR (”Curr Opin Psychiatry“[Journal]) OR (”Curr Probl Surg“[Journal]) OR (”Curr Psychiatry Rep“[Journal]) OR (”Curr Rev Musculoskelet Med“[Journal]) OR (”Diabetes Technol Ther“[Journal]) OR (”Diabetes Metab Res Rev“[Journal]) OR (”Diagn Interv Imaging“[Journal]) OR (”Dialogues Clin Neurosci“[Journal]) OR (”Dig Endosc“[Journal]) OR (”Drug Deliv Transl Res“[Journal]) OR (”Drug Des Devel Ther“[Journal]) OR (”Drug Resist Updat“[Journal]) OR (”Ear Hear“[Journal]) OR (”Ecotoxicol Environ Saf“[Journal]) OR (”Emerg Microbes Infect“[Journal]) OR (”Environ Health“[Journal]) OR (”Environ Health Prev Med“[Journal]) OR (”Environ Health Perspect“[Journal]) OR (”Environ Toxicol Pharmacol“[Journal]) OR (”Epidemiol Psychiatr Sci“[Journal]) OR (”Euro Surveill“[Journal]) OR (”Eur Arch Psychiatry Clin Neurosci“[Journal]) OR (”Eur Cell Mater“[Journal]) OR (”Eur Child Adolesc Psychiatry“[Journal]) OR (”Eur Heart J Cardiovasc Imaging“[Journal]) OR (”Eur Heart J Cardiovasc Pharmacother“[Journal]) OR (”Eur J Cardiothorac Surg“[Journal]) OR (”Eur J Cardiovasc Nurs“[Journal]) OR (”Eur J Nucl Med Mol Imaging“[Journal]) OR (”Eur J Phys Rehabil Med“[Journal]) OR (”Eur J Psychotraumatol“[Journal]) OR (”Eur J Surg Oncol“[Journal]) OR (”Eur J Vasc Endovasc Surg“[Journal]) OR (”Eur Neuropsychopharmacol“[Journal]) OR (”Eur Psychiatry“[Journal]) OR (”Eur Rev Aging Phys Act“[Journal]) OR (”Expert Opin Drug Deliv“[Journal]) OR (”Expert Opin Drug

© 2026 Ren Y et al. *JAMA Network Open*.

Discov“[Journal]) OR (”Expert Opin Drug Metab Toxicol“[Journal]) OR (”Expert Opin Investig Drugs“[Journal]) OR (”Expert Opin Ther Targets“[Journal]) OR (”Expert Rev Anti Infect Ther“[Journal]) OR (”Eye Vis (Lond)“[Journal]) OR (”Eye (Lond)“[Journal]) OR (”Forensic Sci Int Genet“[Journal]) OR (”Free Radic Biol Med“[Journal]) OR (”Front Endocrinol (Lausanne)“[Journal]) OR (”Front Integr Neurosci“[Journal]) OR (”Front Neuroendocrinol“[Journal]) OR (”Front Med“[Journal]) OR (”Gen Hosp Psychiatry“[Journal]) OR (”Genes Dev“[Journal]) OR (”Genomics Proteomics Bioinformatics“[Journal]) OR (”Global Health“[Journal]) OR (”Head Neck“[Journal]) OR (”Health Aff (Millwood)“[Journal]) OR (”Health Res Policy Syst“[Journal]) OR (”Hear Res“[Journal]) OR (”Hepatobiliary Surg Nutr“[Journal]) OR (”Hortic Res“[Journal]) OR (”IEEE J Biomed Health Inform“[Journal]) OR (”IEEE Trans Med Imaging“[Journal]) OR (”IEEE Trans Neural Syst Rehabil Eng“[Journal]) OR (”Infect Dis (Lond)“[Journal]) OR (”Intensive Crit Care Nurs“[Journal]) OR (”Intensive Care Med“[Journal]) OR (”Int Forum Allergy Rhinol“[Journal]) OR (”Int J Antimicrob Agents“[Journal]) OR (”Int J Clin Health Psychol“[Journal]) OR (”Int J Health Policy Manag“[Journal]) OR (”Int J Hyg Environ Health“[Journal]) OR (”Int J Lang Commun Disord“[Journal]) OR (”Int J Ment Health Nurs“[Journal]) OR (”Int J Nanomedicine“[Journal]) OR (”Int J Pharm“[Journal]) OR (”Int J Radiat Oncol Biol Phys“[Journal]) OR (”Int J Transgend Health“[Journal]) OR (”Invest Ophthalmol Vis Sci“[Journal]) OR (”Isr J Health Policy Res“[Journal]) OR (”JACC Basic Transl Sci“[Journal]) OR (”JACC Cardiovasc Imaging“[Journal]) OR (”JACC Cardiovasc Interv“[Journal]) OR (”JACC Clin Electrophysiol“[Journal]) OR (”JAMA Otolaryngol Head Neck Surg“[Journal]) OR (”JAMA Psychiatry“[Journal]) OR (”JAMA“[Journal]) OR (”JMIR Public Health Surveill“[Journal]) OR (”J Affect Disord“[Journal]) OR (”J Appl Res Intellect Disabil“[Journal]) OR (”J Cachexia Sarcopenia Muscle“[Journal]) OR (”J Cardiovasc Comput Tomogr“[Journal]) OR (”J Cardiovasc Magn Reson“[Journal]) OR (”J Cereb Blood Flow Metab“[Journal]) OR (”J Child Psychol Psychiatry“[Journal]) OR (”J Clin Child Adolesc Psychol“[Journal]) OR (”J Community Health“[Journal]) OR (”J Consult Clin Psychol“[Journal]) OR (”J Craniomaxillofac Surg“[Journal]) OR (”J Endod“[Journal]) OR (”J Epidemiol Community Health“[Journal]) OR (”J Epidemiol Glob Health“[Journal]) OR (”J Exp Clin Cancer Res“[Journal]) OR (”J Expo Sci Environ Epidemiol“[Journal]) OR (”J Health Polit Policy Law“[Journal]) OR (”J Infect Public Health“[Journal]) OR (”J Integr Med“[Journal]) OR (”J Intellect Disabil Res“[Journal]) OR (”J Investig Allergol Clin Immunol“[Journal]) OR (”J Learn Disabil“[Journal]) OR (”J Microbiol Immunol Infect“[Journal]) OR (”J Midwifery Womens Health“[Journal]) OR (”J Minim Invasive Gynecol“[Journal]) OR (”J Neuroimmune Pharmacol“[Journal]) OR (”J Neuroinflammation“[Journal]) OR (”J Neurol Neurosurg Psychiatry“[Journal]) OR (”J Neurosurg Anesthesiol“[Journal]) OR (”J Nurs Manag“[Journal]) OR (”J Nurs Scholarsh“[Journal]) OR (”J Occup Health Psychol“[Journal]) OR (”J Orthop Translat“[Journal]) OR (”J Otolaryngol Head Neck Surg“[Journal]) OR (”J Pain Symptom Manage“[Journal]) OR (”J Parkinsons Dis“[Journal]) OR (”J Pediatr Health Care“[Journal]) OR (”J Psychiatr Ment Health Nurs“[Journal]) OR (”J Public Health Policy“[Journal]) OR (”J Public Health (Oxf)“[Journal]) OR (”J Speech Lang Hear Res“[Journal]) OR (”J Sports Sci“[Journal]) OR (”J Am Acad Child Adolesc Psychiatry“[Journal]) OR (”J Am Coll Surg“[Journal]) OR (”J Am Med Inform Assoc“[Journal]) OR (”J Am Soc Echocardiogr“[Journal]) OR (”J Eur Acad Dermatol Venereol“[Journal]) OR (”J Int Soc Sports Nutr“[Journal]) OR (”J Natl Cancer Inst“[Journal]) OR (”J Natl Compr Canc Netw“[Journal]) OR (”J R Soc Med“[Journal]) OR (”J Toxicol Environ Health B Crit Rev“[Journal]) OR (”J Vasc Surg Venous Lymphat Disord“[Journal]) OR (”J Wound Ostomy Continence Nurs“[Journal]) OR (”Knee Surg Sports Traumatol Arthrosc“[Journal]) OR (”Lang Speech Hear Serv Sch“[Journal]) OR (”Liver Transpl“[Journal]) OR (”Med Sci Sports Exerc“[Journal]) OR (”Mil Med Res“[Journal]) OR (”MMWR Morb Mortal Wkly Rep“[Journal]) OR (”Mob DNA“[Journal]) OR (”Mod Pathol“[Journal]) OR (”Mol Pharm“[Journal]) OR (”Mol Psychiatry“[Journal]) OR (”Mol Ther Nucleic Acids“[Journal]) OR (”Mov Disord“[Journal]) OR (”Mult Scler“[Journal]) OR (”Mutat Res Rev Mutat Res“[Journal]) OR (”Nat Rev Gastroenterol Hepatol“[Journal]) OR (”Nephrol Dial Transplant“[Journal]) OR (”Network“[Journal]) OR (”Neural Netw“[Journal]) OR (”Neurol Neuroimmunol Neuroinflamm“[Journal]) OR (”Neuropathol Appl Neurobiol“[Journal]) OR (”Neuropsychopharmacology“[Journal]) OR (”Neurorehabil Neural Repair“[Journal]) OR (”Neurosci Biobehav Rev“[Journal]) OR (”Nurse Educ Today“[Journal]) OR (”Nurse Educ“[Journal]) OR (”Obesity (Silver Spring)“[Journal]) OR (”Occup Med (Lond)“[Journal]) OR (”One Health“[Journal]) OR (”Osteoarthritis Cartilage“[Journal]) OR (”Otolaryngol Head Neck Surg“[Journal]) OR (”Paediatr Drugs“[Journal]) OR (”Palliat Med“[Journal]) OR (”Parasit Vectors“[Journal]) OR (”Pediatr

© 2026 Ren Y et al. *JAMA Network Open*.

Allergy Immunol“[Journal]) OR (”Pediatr Crit Care Med“[Journal]) OR (”Perspect Public Health“[Journal]) OR (”Pharmacol Ther“[Journal]) OR (”Pharmacol Biochem Behav“[Journal]) OR (”Pigment Cell Melanoma Res“[Journal]) OR (”PLoS Negl Trop Dis“[Journal]) OR (”Prog Neuropsychopharmacol Biol Psychiatry“[Journal]) OR (”Prostate Cancer Prostatic Dis“[Journal]) OR (”Psychiatr Serv“[Journal]) OR (”Psychiatry Clin Neurosci“[Journal]) OR (”Psychiatry Res“[Journal]) OR (”Psychol Trauma“[Journal]) OR (”Psychol Res Behav Manag“[Journal]) OR (”QJM“[Journal]) OR (”Reg Anesth Pain Med“[Journal]) OR (”Regul Toxicol Pharmacol“[Journal]) OR (”Reprod Biol Endocrinol“[Journal]) OR (”Respir Res“[Journal]) OR (”Rev Endocr Metab Disord“[Journal]) OR (”Rev Environ Contam Toxicol“[Journal]) OR (”Braz J Psychiatry“[Journal]) OR (”Scand J Med Sci Sports“[Journal]) OR (”Scand J Work Environ Health“[Journal]) OR (”Schizophr Bull“[Journal]) OR (”Sci Med Footb“[Journal]) OR (”Sex Reprod Health Matters“[Journal]) OR (”SSM Popul Health“[Journal]) OR (”Telemed J E Health“[Journal]) OR (”Am J Bioeth“[Journal]) OR (”Am J Chin Med“[Journal]) OR (”Am J Geriatr Psychiatry“[Journal]) OR (”Am J Psychiatry“[Journal]) OR (”Am J Sports Med“[Journal]) OR (”Br J Dermatol“[Journal]) OR (”Br J Gen Pract“[Journal]) OR (”Br J Ophthalmol“[Journal]) OR (”Br J Psychiatry“[Journal]) OR (”Br J Surg“[Journal]) OR (”Cochrane Database Syst Rev“[Journal]) OR (”Int J Behav Nutr Phys Act“[Journal]) OR (”Int J Eat Disord“[Journal]) OR (”Int J Neuropsychopharmacol“[Journal]) OR (”Int J Soc Psychiatry“[Journal]) OR (”J Adolesc Health“[Journal]) OR (”J Allergy Clin Immunol“[Journal]) OR (”J Allergy Clin Immunol Pract“[Journal]) OR (”J Antimicrob Chemother“[Journal]) OR (”J Clin Endocrinol Metab“[Journal]) OR (”J Clin Psychiatry“[Journal]) OR (”J Evid Based Dent Pract“[Journal]) OR (”J Head Trauma Rehabil“[Journal]) OR (”J Heart Lung Transplant“[Journal]) OR (”J Infect“[Journal]) OR (”J Orthop Sports Phys Ther“[Journal]) OR (”J Pediatr“[Journal]) OR (”J Physiol“[Journal]) OR (”J Prev Alzheimers Dis“[Journal]) OR (”J Am Acad Orthop Surg“[Journal]) OR (”J Thorac Cardiovasc Surg“[Journal]) OR (”J Trauma Acute Care Surg“[Journal]) OR (”J Urol“[Journal]) OR (”J Gerontol B Psychol Sci Soc Sci“[Journal]) OR (”Lancet Reg Health West Pac“[Journal]) OR (”Lancet Child Adolesc Health“[Journal]) OR (”Lancet Diabetes Endocrinol“[Journal]) OR (”Lancet Gastroenterol Hepatol“[Journal]) OR (”Lancet Psychiatry“[Journal]) OR (”Lancet Respir Med“[Journal]) OR (”Med Clin North Am“[Journal]) OR (”Med J Aust“[Journal]) OR (”N Engl J Med“[Journal]) OR (”Plant Genome“[Journal]) OR (”Proc Nutr Soc“[Journal]) OR (”Surg Clin North Am“[Journal]) OR (”Ther Adv Neurol Disord“[Journal]) OR (”Thromb Haemost“[Journal]) OR (”Tissue Eng Part B Rev“[Journal]) OR (”Transl Psychiatry“[Journal]) OR (”Travel Med Infect Dis“[Journal]) OR (”Trends Cardiovasc Med“[Journal]) OR (”Trends Endocrinol Metab“[Journal]) OR (”Ultrasound Obstet Gynecol“[Journal]) OR (”United European Gastroenterol J“[Journal]) OR (”Wiley Interdiscip Rev Nanomed Nanobiotechnol“[Journal]) OR (”Worldviews Evid Based Nurs“[Journal]) OR (”Except Child“[Journal]) OR (”Journal of sport management“[Journal]) OR (”Mater Sci Eng C Mater Biol Appl“[Journal]) OR (”Clin Psychol (New York)“[Journal]) OR (”Res Pract Persons Severe Disabl“[Journal]) OR (”Int J Ment Health Addict“[Journal]) OR (”Res Autism Spectr Disord“[Journal]) OR (”J Drug Deliv Sci Technol“[Journal]) OR (”Cognit Comput“[Journal]) OR (”Rev Psiquiatr Salud Ment“[Journal]) OR (”J Ment Health Res Intellect Disabil“[Journal]) OR (”Wiley Interdiscip Rev Syst Biol Med“[Journal]) OR (”J Pharm Investig“[Journal]) OR (”Biodes Manuf“[Journal]) OR (”Career Dev Transit Except Individ“[Journal]) OR (”Health Policy Technol“[Journal]) OR (”J Contextual Behav Sci“[Journal]) OR (”Anal Methods Accid Res“[Journal]) OR (”Int J Bioprint“[Journal]) OR (”Food reviews international“[Journal]) OR (”Food science and human wellness“[Journal])

7. #5 AND #6

© 2026 Ren Y et al. *JAMA Network Open*.

eAppendix 2. Automated screening using the fPIS-ML technique

We used a pre-designed machine learning–assisted framework—fuzzy Population–Intervention–Study design (fPIS) combined with machine learning technique—to assist in identifying eligible TTE studies.

Retrieve all potential related records from database

We conducted a search for TTE-related literature in the PubMed database using the text terms "target*", "emulat*" and "trial*", and restricted the search to articles published between January 1, 2017 and December 15, 2023. After deduplication processing, a total of 46,986 retrieval records were obtained. The full-text screening process was applied to a total of 40,637 records.

Step 1: Accelerate data labeling by expert-formulated rules

We applied a keyword co-occurrence rule to screen for possible TTE literature. The rule stipulates that key1 and key2 should appear in a same sentence, and the distance between them should be no more than 10 words, where key1 includes: 'trial*', and key2 includes: 'emulat*','mimic*', 'simulat*', the specific keywords are shown in Table 1. For an article, if it meets this co-occurrence rule in the full-text, it is a candidate; otherwise, it will be excluded directly.

Then, the candidates are manually screened, independently evaluated by two field experts, and the third arbitrator resolves the differences to determine clear inclusion and exclusion. Finally, a total of 593 articles (gold standard dataset) were labeled, of which 68 were TTE (positive samples). This dataset was called D and used for model development and validation.

eAppendix 2.Table 1. The keywords of rules for TTE.

key 1	key 2
'trial', 'trials'	'emulate', 'emulated', 'emulating', 'emulation', 'emulates','emulat','mimic', 'mimicking', 'simulate', 'simulated', 'simulating', 'simulation', 'simulates','simulat'

© 2026 Ren Y et al. *JAMA Network Open*.

Step 2: Develop a prediction model for identifying target literature

Dataset ***D*** was divided into the development dataset and the internal validation dataset at a ratio of 8:2. For external validation, we collected and collated TTE literature from three TTE reviews reporting from the journals BMC Medical Research Methodology [1], JAMA Network Open [2] and Journal of Clinical Epidemiology [3]. Data deduplication was performed with the retrieved literature above, and 175 TTE literature was collected as the external validation dataset.

We implemented six machine learning models and evaluated their performance, including four commonly methods (Logistic Regression (LR), Naive Bayes (NB), Support Vector Machine (SVM) and Neural Network (NN)) and two bagging or boosting ensemble learning models (Random Forest (RF) and Gradient Boosting Decision Tree (GBDT)). Five-fold cross-validation was used for each algorithm.

For the internal validation, among the six ML models, GBDT based on gradient boosting, performed significantly better than the other models, and GBDT achieves the optimal performance with 92.86% F1-score, 92.86% sensitivity and 99.05% specificity (Figure 1). For external validation, GBDT also showed the best balance recognition results compared to the other models, with an accuracy of 88.57% (Figure 2). Based on the internal and external validation results, GBDT comprehensively showed the best balance performance. **Therefore, we chose GBDT as the final model for TTE identification.**

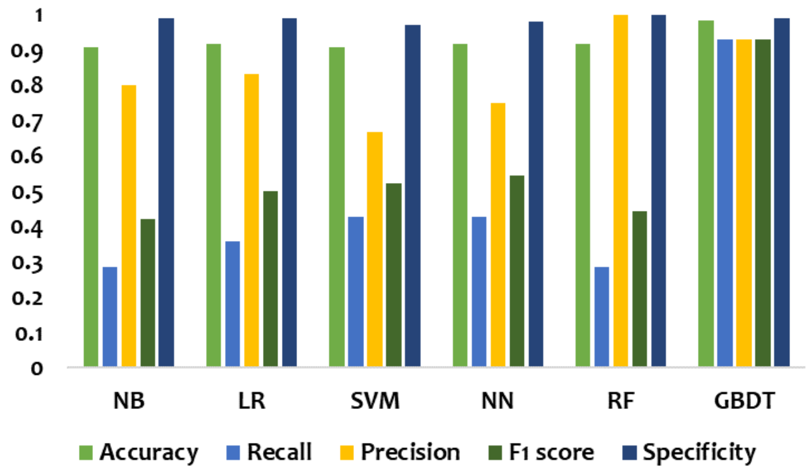

© 2026 Ren Y et al. *JAMA Network Open*.

eAppendix 2.Figure 1. The performance of each ML model on internal validation dataset

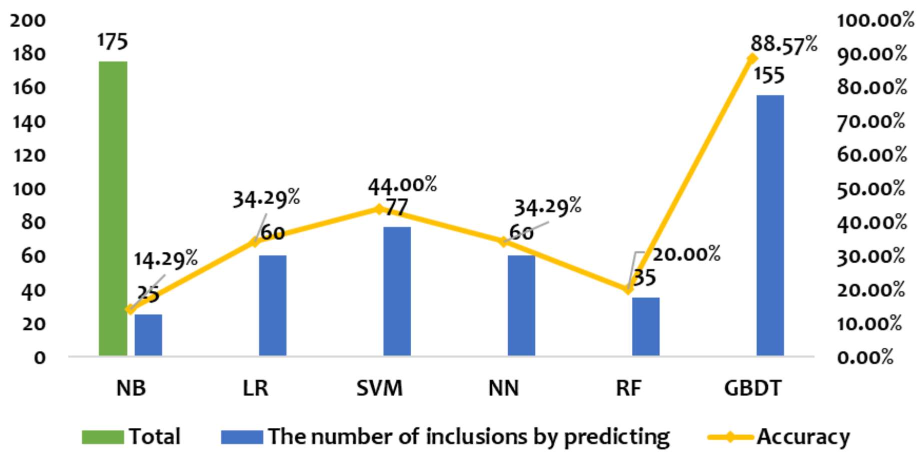

eAppendix 2.Figure 2. The results of each ML model on external validation dataset

Step3 GBDT model for predicting target literature

The remaining 40044 literature from Step 2 (minus the modelling data) was inputted into the trained 5-fold GBDT model for prediction. Each fold of the trained model was considered as a voter, so there were five votes for each piece of literature. We performed manual verification in varying proportions for different cases of voting. When the number of votes = 0, we randomly selected 500 literatures for manual verification, and all other cases (≥3, =1, =2) were manually screened. The results sampled by the number of votes = 0 were all excluded after manual verification, which matches well with the high specificity of the model. About 85% of the literature with ≥3 votes were target TTE, which indicated that the results are reliable when the number of votes is more than half.

Reference

[1] Scola G, Chis Ster A, Bean D, et al. Implementation of the trial emulation approach in medical research: a scoping review. BMC Med Res Methodol. 2023;23:186. doi: 10.1186/s12874-023-02000-9

[2] Hansford HJ, Cashin AG, Jones MD, et al. Reporting of Observational Studies Explicitly Aiming to Emulate Randomized Trials: A Systematic Review. JAMA Netw Open. 2023;6:e2336023. doi:

© 2026 Ren Y et al. *JAMA Network Open*.

10.1001/jamanetworkopen.2023.36023

[3] Zuo H, Yu L, Campbell SM, et al. The implementation of target trial emulation for causal inference: a scoping review. J Clin Epidemiol. 2023;162:29–37. doi: 10.1016/j.jclinepi.2023.08.003

© 2026 Ren Y et al. *JAMA Network Open*.

eAppendix 3. Data abstraction form

Ref ID: ________________________

Author (first author's last name): ________________________

Journal name _______________________

Year of Publication: ________________________

☐ Is the study published on one of the high impact journals (NEJM, JAMA, LANCET, BMJ)?

Country of corresponding author _______________________

SECTION 1: General characteristics

1.1 What is the study design?

Illustration: If the real study design is not consistent with author's statement, please select the real design type

☐ Prospective cohort study
☐ Retrospective cohort study
☐ Case-control study
☐ Other, please specify
☐ Not reported

1.2 What kind of data sources were used? (Check all that apply)

Illustration: Select all kinds claimed by the author

☐ Registry
☐ Administrative claims database
☐ Electronic Medical Records
☐ Traditional epidemiological survey
☐ Surveillance database
☐ Others, please specify_____
☐ Not reported

1.3 The coverage of the data source

☐ One center or hospital
☐ International center
☐ National center
☐ Regional center

1.4 Sample size eligible: _____, Sample size for primary analysis: _____

Illustration: If the study emulated more than one trial, reported the sample size of first reported emulated trial; If PSM was performed, the sample size eligible was the pre-PSM sample size, and the sample size for the primary analysis was the post-PSM sample size.

1.5 Is there a methodologist involved?

Illustration: (1) Author Affiliations: including Department of epidemiology or statistics or public health; (2) Acknowledgment: The authors thank someone for statistical support; (3) invited the methodologists (epidemiology or statistics) to attend this study.

☐ Yes
☐ No

© 2026 Ren Y et al. *JAMA Network Open*.

1.6 What are the sources of funding? (check all that apply)

Illustration: Both partial and complete funding must be reported; if at least one of the team members is funded, please select funded. When the nature of the funding agency is not clear, google it.

- ☐ Government funding: e.g.; NIH (national institutes for health), CIHR (Canadian Institutes of Health Research)
- ☐ Private for profit: e.g., drug companies, device companies
- ☐ Private not for profit: e.g., foundations, not for profit universities, not for profit hospitals
- ☐ No funding: the study should explicitly say it.
- ☐ Not reported: if no information provided

SECTION 2: PICO (Population, Intervention, Control, Outcome) characteristics

2.1 Whether specified the target disease？

Illustration: The classification criterion for "Target disease, No," was based on the description of the target population in the Methods section and indicates that no specific disease type was reported. Study participants in this category may include healthy individuals (e.g., samples from the general population), patients prescribed specific medications (e.g., statin initiators versus non-initiators), or individuals receiving particular interventions (e.g., hemodialysis four times per week compared to three times per week).

- ☐ Yes
- ☐ No

2.1.1 IF yes, please specify: ________________

2.1.2 IF yes. what are the target diseases?

- ☐ Infectious diseases (COVID-19, HIV, et al)
- ☐ Cardiology (cardiovascular diseases)
- ☐ Oncology (Cancer, et al)
- ☐ Endocrinology (Diabetes, et al)
- ☐ Neurology (cerebrovascular diseases)
- ☐ Rheumatology
- ☐ Psychiatry
- ☐ Psychiatry
- ☐ Kidney disease
- ☐ Orthopedic diseases
- ☐ Obstetrics and Gynecology
- ☐ Critical care medicine
- ☐ Other, please specify:

2.2 Type of intervention

- ☐ Drugs
- ☐ Vaccine
- ☐ Surgical
- ☐ Behavioral intervention

© 2026 Ren Y et al. *JAMA Network Open*.

☐ Other, please specify:

2.3 Type of control (Check all that apply)

Illustration: Blank control indicates that the primary comparison made in the study involved comparing individuals who initiated specific medications with those who did not. The distinction between "blank control" and "standard care" depends on whether the researchers explicitly stated that the control group would receive standard care.

☐ Standard care

☐ Blank control

☐ Active control

☐ Other, please specify:

2.4 Primary outcome, please specify: ________________ (check all that apply)

Illustration: Symptoms/Quality of life/Functional status (e.g. pain, depression, failure to become pregnant, successful nursing/breastfeeding, work disability). If the primary outcome is explicitly reported, all corresponding outcome types are selected. If it is not explicitly specified, the first outcome listed in the abstract is designated as the primary outcome type.

Mortality

☐ All-cause mortality

☐ Disease specific mortality

Morbidity

☐ Cardiovascular major morbid events

☐ Other major morbid events (e.g. Loss of vision, seizures, fracture, revascularization)

☐ Recurrence/relapse/remission of cancer and other chronic diseases (e.g. COPD exacerbation)

☐ Hospitalization

☐ Infections

☐ Symptoms/Quality of life/Functional status

☐ Laboratory examination

☐ Other

2.5 Type of the primary outcome (check all that apply)

☐ Continuous

☐ Ordinal

☐ Binary

☐ Time-to-event

☐ Other, please specify: _____________

SECTION 3: Clinical characteristics

3.1 What does the study aim to evaluate?

☐ Effectiveness

☐ Safety

☐ Safety + effectiveness

☐ Cost-Effectiveness

3.2 Which scenario did the emulated trial present? (check all that apply)

☐ Replicating the results of a published trial in real world clinical setting

© 2026 Ren Y et al. *JAMA Network Open*.

- ☐ Estimating the effectiveness or safety for previous conflicting results reported
- ☐ Expanding the intervention population for ethical reasons
- ☐ Estimating the comparative effectiveness for active control
- ☐ Estimating the effectiveness for treatment switching
- ☐ Estimating the effectiveness or safety for long-term outcome
- ☐ Estimating the safety for rare event outcome
- ☐ Estimating the effectiveness or safety for rare diseases
- ☐ Estimating the effectiveness or safety for emergency medication (e.g., COVID-19 drug)
- ☐ Predicting the results of the RCTs in advance
- ☐ Lacking RCT evidence
- ☐ Estimating effectiveness or safety in real world clinical setting
- ☐ Other, specify: ____

© 2026 Ren Y et al. *JAMA Network Open*.

Illustration for each scenario did the emulated trial present

**Which scenario did the emulated trial present?**	**Annotation**	**Examples**
Replicating the results of a published trial in real world clinical setting	Studies described the intent of comparing TTE and RCT	"Finally, we compared these estimated changes in ASCVD risk with corresponding observed changes in REDUCE-IT and STRENGTH."
Estimating the previous conflicting results reported in studies	Studies described previous studies with inconsistent/conflicting/controversial findings.	"While the prehabilitation concept appears promising, there is conflicting scientific evidence for its effectiveness. Several RCTs have been performed, showing different effects of multimodal prehabilitation, ranging from meaningful changes in postoperative functional walking capacity and significantly improved postoperative clinical outcomes, to no effect on postoperative outcomes"
Estimating the results on broader population	Studies described the inclusion of other ethnic groups or vulnerable populations (e.g., those with comorbidities, the elderly) that were not covered in previous RCTs.	"While some evidence supports statin use for primary prevention of ASCVD in patients with moderate CKD, few older adults were included in clinical trials, and data to guide initiation of a statin in older patients with CKD without prior history of ASCVD are needed."
Estimating the comparative effectiveness for active control	Studies described the intent of comparing the effectiveness of a specific treatment with the standard treatment recommended by guidelines, or of comparing the effectiveness among different positive drugs—comparisons that were not conducted in previous RCTs.	"In RCTs with intention-to-treat analyses (ITT), the different bDMARDs in combination with methotrexate (MTX) appear to have similar efficacy, (2-4) but only some of the RCTs have performed direct comparison of different bDMARDs."<br>"Head-to-head comparison of FOLFIRINOX versus gemcitabine plus nab-paclitaxel in advanced pancreatic cancer: a target trial emulation using real-world data"

© 2026 Ren Y et al. *JAMA Network Open*.

Estimating the effectiveness for treatment switching	Studies described evaluating the effects of switching from one treatment to another.	"The aim of this study was to assess the comparative effectiveness of GP2017 versus SB5 following a mandatory switch regarding 1-year treatment retention rates, 6-month disease remission rates and reasons for withdrawal. In addition, to investigate changes in disease activity 6 months prior to and after the switch, and the frequency and reasons for back-switching to originator adalimumab."
Estimating the effectiveness or safety for long-term outcome	The definition of “long-term” outcome was based on how it was described in the original TTE studies. Most of these studies explicitly highlighted the lack of long-term evidence as a rationale for conducting the emulation, since RCTs in the same area had typically focused on short-term outcomes. Accordingly, outcomes were classified as “long-term” when the investigators emphasized this evidence gap and selected endpoints beyond the usual short-term trial horizon—for example, overall mortality or adverse events emerging over extended follow-up, such as cardiovascular events or cancer risk associated with antidiabetic drug use.	①"Randomized controlled trials did not directly compare efficacy of antiviral agents, nor did they evaluate outcomes beyond 29 days after symptomatic infection."<br>②The aim of this study was to emulate a clinical trial that assesses the long-term relation compliance with the DASH dietary pattern and risk of cardiovascular and all-cause mortality in patients with a history of myocardial infarction (MI).
Estmatig the safety for rare event outcome	Rare event outcomes refer to study outcomes that occur with extremely low frequency in a given population, typically characterized by a low incidence rate (e.g., events affecting less than 5% of the study cohort)	①"The gold standard for estimating the risk of PcP would be to conduct a randomized trial. However, due to the low levels of PcP diagnoses for patients on ART, a randomised trial would be prohibitive both in terms of time and cost."<br>②"However, given the rarity of anaphylactic events, clinical trials—or meta-analyses of such trials—are not adequately powered to detect differences in anaphylaxis among formulations."

© 2026 Ren Y et al. *JAMA Network Open*.

Estimating the effectiveness or safety for rare diseases	Studies described that the disease was rare or had an extremely low incidence rate.	"Duchenne muscular dystrophy (DMD) is an X-linked genetic disorder that affects approximately 1 in every 3500 live-born male infants"<br>"such a trial is not ongoing and unlikely to be accrued quickly given the relative rarity of pN1 disease."
Estimating the effectiveness or safety for emergency medication (e.g. COVD-19 drug)	Studies described that the treatments were emergency medications, or the urgent need for research to generate findings that guide practice (e.g., COVID-19).	"an unprecedented worldwide search for effective treatments. The emergence of highly transmissible variants,1–6 along with evidence of waning vaccine effectiveness,7–12 suboptimal vaccine uptake,13 and reduced vaccine protection against emerging variants14,15 only increased the need for effective and accessible treatments for COVID-19 patients"
Predicting the results of the RCTs in advance	Studies described that research was conducted using real-world data before the publication of RCT results to predict clinical trial outcomes.	"To ascertain whether routinely available claims data can be used to emulate a prospective randomized clinical trial ahead of its publication, filling important methodologic and regulatory policy needs in the use of real-world data to predict clinical trial results."
Lacking RCT evidence	Studies described that RCT evidence was infeasible or unavailable.	"Without data from randomized controlled trials yet, we aimed to emulate a randomized controlled trial with observational data using a causal inference study design."
Estimating effectiveness or safety in real world clinical setting	Studies described limitations of RCTs in sample size, restrictive inclusion criteria, or highly controlled settings, and emphasized the need for studies in real-world settings with a large population.	"Given that individuals included in RCTs may not reflect the veteran population seeking clinical services, it is important to better understand whether veterans who receive CPT or PE can improve in the 'real-world' clinical setting"

© 2026 Ren Y et al. *JAMA Network Open*.

Other, specify.	e.g., ①different region, ②outcomes different from RCTs ③Using published RCT data to study different problems...	①To our knowledge, studies from the UK have hitherto analysed data from a single country, e.g. Scotland9 or England,11 with no multination studies across the UK. The aim of this study was to investigate waning in VE/relative VE (rVE) against severe COVID-19 outcomes using pooled data from across the four nations of the UK using a target trial approach.<br>②"The benefit of ACEi on major kidney outcomes after acute kidney injury requiring short-term dialysis has also been observed (19). However, whether initiation of a RASi after an episode of sepsis reduces the incidence of subsequent cardiovascular complications remains unknown"<br>③Using an active-comparator, new-user design as a secondary analysis of the Systolic Blood Pressure Intervention Trial (SPRINT), we sought to emulate a target trial12 to compare the risk of developing MCI and probable dementia (PD) between those initiating an ARB vs ACEI.

© 2026 Ren Y et al. *JAMA Network Open*.

3.3 What types of treatment strategies the target trial emulation focused on?

☐ Point treatment strategies

☐ Static sustained treatment strategies

☐ Dynamic sustained treatment strategies

3.4 What types of treatment strategies did the target trial emulation compare? (Check all that apply)

☐ Head-to-head comparison of two or more active treatments (drug A vs. drug B)

☐ Single active treatment versus no treatment (drug A vs. no)

☐ Combination treatment versus one active treatment (drug A + drug B vs. drug A)

☐ Combination treatment versus no treatment (drug A + drug B vs.no)

☐ Other, please specify: _____

SECTION 4: Methodology characteristics for designing a target trial

4.1 Has the research protocol been registered?

Illustration: If any registered research protocol was mentioned in the article, please select yes

☐ Yes

☐ No

4.2 Whether made a target trial protocol?

☐ Yes

☐ No

4.3 How many target trials were emulated in this study?

☐ 1

☐ 2

☐ 3 or more, please specify: _____

4.3.1 If more than one target trial was emulated, what was the reason? please specify: _____ (Check all that apply)

☐ Different intervention

☐ Different primary outcome

☐ Different population

☐ Other, please specify: _____

4.4 How many arms were compared in target trials?

☐ 1

☐ 2

☐ 3

☐ 4

☐ More than 4

☐ Not reported

4.5 Which type of target trial was planned to emulate?

Illustration: "Hypothetical RCT" refers to cases where the TTE study explicitly described the target trial as hypothetical. "May be hypothetical" indicates that while the target trial was described, it was not explicitly stated the target trial was based on an existing RCT or hypothetical trial. "Not specified" was selected if the target trial was not explicitly introduced.

☐ Existing complete RCT

© 2026 Ren Y et al. *JAMA Network Open*.

☐ Existing ongoing RCT
☐ Hypothetical RCT
☐ May be Hypothetical RCT
☐ Not specified

If it was an existing RCT, did the researcher give the reason for selecting this RCT as the target trial?

☐ Yes
☐ No

If it was a hypothetical RCT, what would be the reference standards for defining such a trial?

☐ Existing RCT for another primary outcome
☐ Other, specify: _____
☐ Not specified

4.6 Which type RCT was emulated?

☐ Parallel RCT
☐ Cluster RCT
☐ RCT using a hybrid control arm design
☐ Pragmatic trial
☐ Other, please specify:
☐ Not specified

SECTION 5: Methodological characteristics for implementing a TTE study

TTE reporting

5.1 Did the study report following any methodological guideline? (check all that apply)

☐ STROBE
☐ RECORD
☐ ISPOR
☐ Others, please specify:
☐ Not reported

5.2 How the protocol of the emulated target trial was reported?

☐ TTE framework tabular form
☐ TTE framework txt form
☐ Text form not following TTE framework

5.3 What components of target trial emulation were reported? (Check all that apply)

Illustrate: This item refers to the reporting of components in the observational emulation, not in the conceptual target trial specification.

☐ Eligibility criteria
☐ Treatment strategies
☐ Assignment procedures
☐ Outcome(s)
☐ Follow-up
☐ Causal contrast of interest
☐ Statistical methods

© 2026 Ren Y et al. *JAMA Network Open*.

Eligibility criteria

5.4 Whether the eligibility criteria in emulated trial are same as those in target trial?

☐ Yes

☐ No

☐ Not reported

5.5 Whether the eligibility criteria are based only on values that are available at baseline, never on post-baseline information used to define eligibility (e.g., requiring ≥1 follow-up)?

Illustrate: The eligibility criteria cannot include restrictions based on postbaseline events (e.g., "include only individuals who ever used therapy during the follow-up", "patient must be alive for 1 year after study entry"), which may introduce bias in the analysis of both randomized trials and observational data.

☐ Yes

☐ No

5.6 Whether the study specified eligibility patients at multiple time?

Illustrate: An explicit emulation of a target trial was able to reproduce the non-monotonic effect estimates and, when combined with multiple eligibility entry points for each individual (who can therefore be considered exposed and unexposed at different times), also reduced the variance of the estimates compared with simpler approaches. Multiple times indicate that researchers choose all eligible times and conduct a sequence of trials at each eligible time.

☐ Yes

☐ No

5.7 Whether a flow chart is used to describe who is eligible for TTE?

☐ Yes

☐ No

Treatment strategies

5.8 Whether the treatment strategies in emulated trial are same as those in target trial?

☐ Yes

☐ No

☐ Not reported

5.9 Whether the treatment strategies consider the active comparator?

☐ Yes

☐ No

5.10 Which design was used in treatment strategies?

Illustrate: The new user design allowed the inclusion of almost all people who initiated denosumab. The prevalent/modified new user design allowed the inclusion of almost all people who had used denosumab, including those who switched to denosumab from an oral bisphosphonate and those who initiated denosumab; these sequences of drug use represent typical clinical practice, 'not applicable' for non-pharmacologic treatment strategies (e.g., surgery or behavioral interventions).

☐ Prevalent user design

☐ New user design

© 2026 Ren Y et al. *JAMA Network Open*.

☐ Prevalent/modified new user design
☐ Other, specify: ______________
☐ Not applicable
☐ Not specified

Assignment procedures

5.11 Did the study emulate random assignment procedures?

Illustration: "random assignment" was provided within the respective TTE articles or the studies stated that adjusted for confounders were done to emulate randomization.

☐ Yes
☐ No

If yes, what methods did the study use to emulate random assignment?

☐ Adjustment (e.g., via IP weighting)
☐ Clone
☐ Other, specify ________________
☐ No methods described

Follow-up

5.12 Whether a design diagram is reported to summarize visually the longitudinal follow-up design aspects of a study?

☐ Yes
☐ No

Outcome(s)

5.13 Was the primary outcome in the emulated trial the same as that in the target trial?

☐ Yes
☐ No
☐ Not reported

5.14 Did the author assess the quality of outcome?

Illustration: The quality of outcome measurement depends on positive predicted value for binary outcomes, proportion missing for continuous outcomes, and accurate onset for time-to-event outcomes.

☐ Yes
☐ No

Causal contrast of interest

5.15 Did the authors explicitly state their causal contrast of interest?

☐ Yes
☐ No

If yes, what was the declared or inferred causal contrast in target trial? (check all that apply)

Illustration: Intention-to-treat effect: participants are analysed according to the treatment strategy with which their data are compatible at baseline. Per protocol effect: the effect that would have been observed under full adherence to

© 2026 Ren Y et al. *JAMA Network Open*.

the assigned treatment strategy. As-treated (AT) effect: This estimates the effect of actually receiving the treatment, by comparing outcomes based on the treatment participants actually received rather than their original assignment.

☐ ITT (intention-to-treat effect)

☐ PP (per protocol effect)

☐ As-treated

If yes, what was the declared or inferred causal contrast in emulated target trial? (check all that apply)

Illustration: Intention-to-treat effect: participants are analysed according to the treatment strategy with which their data are compatible at baseline. Per protocol effect: the effect that would have been observed under full adherence to the assigned treatment strategy. As-treated (AT) effect: This estimates the effect of actually receiving the treatment, by comparing outcomes based on the treatment participants actually received rather than their original assignment.

☐ ITT (intention-to-treat effect)

☐ PP (per protocol effect)

☐ As-treated

Statistical methods

5.16 Whether used the statistical method to assess the potential influence of unmeasured confounders?

☐ Yes

☐ No

5.17 Was sensitivity analysis used to check the robustness of the results?

☐ Yes

☐ No

© 2026 Ren Y et al. *JAMA Network Open*.

eAppendix 4. Expert Consultation Questionnaire

Target Trial Emulation in Observational Researches: Recommendations for Design and Implementation
Expert Consultation Questionnaire

Background

Target trial emulation (TTE) is increasingly used to estimate causal effects using real-world data (RWD). We conducted a cross-sectional methodological survey to identify methodological issues in trial design and implementation.

In this comprehensive methodological review of 237 target trial emulation (TTE) studies published in high-impact clinical journals from 2017 to 2023, we found that typical clinical applications included replicating or predicting results from published trials, estimating the effectiveness or safety of interventions amid conflicting evidence, evaluating long-term or rare outcomes, assessing emergency-use interventions (e.g., COVID-19 treatments), and comparing active treatments in routine care. Time-varying treatment strategies were incorporated in 139 studies (58.6%).

Despite the central importance of clearly specifying a target trial, 134 studies (56.5%) did not develop a target trial protocol, and most failed to justify the choice of an existing versus hypothetical trial. Furthermore, 37 studies designed multiple target trials to accommodate differences in interventions or populations. Regarding implementing, only 35 studies (14.8%) employed sequential trial emulation. Inappropriate use of post-baseline information for eligibility was found in 36 studies (15.2%). Additionally, half of the studies did not consider the use of active comparators. A large proportion failed to emulate random assignment (79, 33.3%), define time zero through a follow-up diagram (197, 83.1%), or address the unmeasured confounding (164, 69.2%).

Although TTE is widely applied across various clinical domains, significant methodological gaps remain in defining target trials and applying core principles. We propose a structured five-step framework and a set of practical considerations to guide future TTE design and implementation.

© 2026 Ren Y et al. *JAMA Network Open*.

Instructions for completing the form

Part I: Your basic information* - *please complete all required fields.

Part II: A Structured Framework for Designing Target Trials

To enhance methodological rigor and improve consistency across TTE studies, we propose a structured five-step framework for designing a target trial that is aligned with the research objective and the underlying clinical context. Please indicate your level of agreement with each step: **agree, partially agree, or disagree**. If you choose partially agree or disagree, please provide your opinion or suggestions.

Part III: Recommendations for Implementing core methodological components in TTE

To increasing reliability in causality from TTE studies, the accurate specification of seven core methodological items is essential. We present recommendations for each item. Please indicate your level of agreement: **agree, partially agree, or disagree**. If you select partially agree or disagree, please provide your opinion or suggestions.

© 2026 Ren Y et al. *JAMA Network Open*.

Part I Your Basic information

**Name**		**Sex**	☐ **Male** ☐**Female**
**Highest Degree**	☐ Bachelor's ☐ Master's ☐ MD ☐ PhD		
**Professional Title**	☐ Professor ☐ Associate Senior ☐ Other (Specify): ____		
**Affiliation**			
**Field(s) of Expertise (multiple selections allowed; please specify years of experience)**			
☐ Epidemiology & Biostatistics, Years of Experience: ________<br>☐ Clinical Medicine, Years of Experience: ________<br>☐ Healthcare Administration, Years of Experience: ________<br>☐ Other (Please Specify): ________________, Years of Experience: ________<br>**Have you ever conducted TTE- or observational studies?**<br>☐ Yes ☐ No			
The following information is collected solely for disbursing consultation allowances and will be kept strictly confidential.			
Full Name			
Mobile Number			
ID or Passport Number			
Bank Card Number			
Bank Branch (Specify Sub-Branch)			
**Conflict of Interest Statement:** ☐ **Yes** ☐ **No**			

© 2026 Ren Y et al. *JAMA Network Open*.

Part II A Structured Framework for Designing Target Trials

Steps	Agree	Partially agree	Disagree
**Step 1: Define the research purpose**<br>A valid TTE must begin with a well-defined research purpose concerning the effect of an intervention.			
If you selected “Partially Agree” or “Disagree”, please specify your reasoning or suggestions:			
**Step 2: Identify the application scenario**<br>It is recommended to begin by clearly identifying the context to which the target trial emulation (TTE) will be applied. TTE application scenarios can generally be categorized into the following three types:<br>• **Scenario 1:** Replicating or predicting findings from existing randomized controlled trials (RCTs) in real-world settings.<br>• **Scenario 2:** Extending the evidence base, such as including broader or underrepresented populations, evaluating rare or long-term outcomes, or assessing treatment-switching strategies.<br>• **Scenario 3:** Addressing questions in ethically constrained settings where RCTs are infeasible.<br>Accurate classification of the scenario is encouraged, as it informs subsequent design decisions and ensures the appropriateness of the emulation approach.			
→ If you selected “Partially Agree” or “Disagree”, please specify your reasoning or suggestions:			
**Step 3: Specify the PICO**<br>Researchers are advised to explicitly define the PICO (Population, Intervention, Comparator, and Outcome) of the hypothetical target trial. A well-specified PICO framework facilitates alignment between the research objective and study design, and enhances clarity and reproducibility.			
→ If you selected “Partially Agree” or “Disagree”, please specify your reasoning or suggestions:			

© 2026 Ren Y et al. *JAMA Network Open*.

**Step 4: Review existing RCTs**<br>Where applicable, it is suggested to consult existing RCTs to guide the development of the target trial protocol:<br>• **For Scenario 1,** emulation should be based on a matched RCT, allowing for comparison or prediction in a real-world population.<br>• **For Scenario 2**, related RCTs may be used to inform the plausibility of key design elements in the hypothetical trial。<br>• **For Scenario 3**, when no RCTs are available, the hypothetical trial should be justified based on clinical rationale, biological plausibility, or expert consensus<br>• Such review enhances the credibility of the target trial and supports the emulation with precedent evidence where available.			
→ If you selected "Partially Agree" or "Disagree", please specify your reasoning or suggestions:			
**Step 5: Specify a Transparent Target Trial Protocol**<br>It is strongly recommended to develop a clear and detailed protocol that includes:<br>• Eligibility criteria<br>• Definitions of interventions and comparator<br>• Follow-up strategies<br>• Outcome definitions<br>• Specification of the causal contrast of interest<br>A transparent protocol ensures methodological consistency and supports evaluation of the internal validity and replicability of the emulated trial.			
→ If you selected "Partially Agree" or "Disagree", please specify your reasoning or suggestions:			

© 2026 Ren Y et al. *JAMA Network Open*.

**Consider a Dual-Protocol Design to Support Scenario 2 Applications**<br>For **Scenario 2**, where the research objective extends beyond the scope of existing randomized controlled trials (RCTs)—such as evaluating new outcomes (e.g., safety), broader populations, or longer follow-up periods—it is recommended to develop **two related target trial protocols** to strengthen methodological transparency and internal consistency.<br>We recommend the following two-step protocol strategy:<br>• **Protocol A (Validation Trial Protocol):** design a target trial that emulates a well-conducted existing RCT (e.g., one focused on efficacy). This emulation should closely align with the original RCT in terms of population, intervention, comparator, outcomes, and follow-up duration. The aim is to verify that the emulated design yields comparable effect estimates—at minimum, demonstrating consistency in the direction of effect. This serves as a methodological benchmark, ensuring that key trial elements are accurately operationalized in the real-world dataset.<br>• **Protocol B (Extension Trial Protocol):** based on the validated design of Protocol A, construct a second target trial addressing the actual research question of interest (e.g., assessing safety outcomes or evaluating effects in underrepresented populations). This protocol may appropriately modify certain parameters such as outcomes, eligibility criteria, or follow-up time, while retaining the structural integrity confirmed in Protocol A. The rationale for these modifications should be explicitly stated and grounded in both clinical reasoning and the findings of Protocol A.<br>This dual-protocol approach is especially valuable when extending beyond the evidence base of existing RCTs. It supports transparent justification of design choices, enhances reproducibility, and strengthens the interpretability of causal inference derived from observational data.			
→ If you selected "Partially Agree" or "Disagree", please specify your reasoning or suggestions:			

© 2026 Ren Y et al. *JAMA Network Open*.

Part III Recommendations for implementing core methodological components in TTE

Items	Agree	Partially agree	Disagree
**1. Eligibility Criteria**			
1.1 We recommend that eligibility criteria be defined strictly based on information available at baseline (i.e., prior to treatment initiation) to avoid post-baseline selection bias.			
→If you choose to partially agree or disagree, please give your opinion or suggestion:			
1.2 In situations where patients may meet eligibility criteria at multiple time points, we recommend considering a sequential trial emulation approach. This can improve statistical power while maintaining alignment with the target trial framework.			
→If you choose to partially agree or disagree, please give your opinion or suggestion:			
**2. Follow-up and Time Zero Definition**			
2.1 We recommend that time zero (i.e., the start of follow-up) be clearly and explicitly defined, with alignment between eligibility assessment and treatment assignment. Misalignment among these elements may occur and could introduce biases such as selection bias, prevalent user bias, and immortal time bias. Appropriate methodological approaches should be employed to address these potential biases.			
→If you choose to partially agree or disagree, please give your opinion or suggestion:			
2.2 It is recommended to consider the use of grace periods before time zero to assess prior medical history, baseline covariates, and registration duration. This approach may help ensure more accurate adjustment for confounding and more appropriate specification of inclusion criteria.			
→If you choose to partially agree or disagree, please give your opinion or suggestion:			
2.3 To improve clarity and reproducibility, we suggest using a graphical timeline or design diagram to visually illustrate the structure of follow-up, especially when eligibility and treatment occur asynchronously.			
→If you choose to partially agree or disagree, please give your opinion or suggestion:			
**3. Outcome Definition and Measurement**			
3.1 It is advisable to prespecify the outcome(s) of interest, including their clinical definitions, time frames, and relevance to the causal question.			
→If you choose to partially agree or disagree, please give your opinion or suggestion:			

© 2026 Ren Y et al. *JAMA Network Open*.

3.2 Data sources used for outcome identification should be clearly documented (e.g., electronic health records, claims data), along with the validation methods and reliability metrics.			
→If you choose to partially agree or disagree, please give your opinion or suggestion:			
3.3 We encourage transparent reporting of the methods used to measure and verify outcomes, including any algorithms or clinical adjudication processes applied.			
If you choose to partially agree or disagree, please give your opinion or suggestion:			
3.4 It is advisable to specify primary/secondary outcome(s) in the case of multiple outcomes			
If you choose to partially agree or disagree, please give your opinion or suggestion:			
**4.Treatment strategies**			
4.1 When feasible, an active-comparator new-user design is recommended to assess the real-world safety and effectiveness of medications; only patients who are the first dispensation recorded at time zero and who are naive to both treatments in the previous grace period are included. This design is largely due to the baseline washout period (mimicking a clinical trial) and using an active comparator to reduce confounding by indication. However, this design does not fully eliminate indication bias, as treatment choices are still influenced by disease severity.			
→If you choose to partially agree or disagree, please give your opinion or suggestion:			
4.2 In modified/prevalent new-user designs—which include both patients previously exposed to the comparator and those who are treatment-naïve—this design may be particularly useful in drug safety evaluations when comparing a newly introduced treatment with a long-established comparator.			
If you choose to partially agree or disagree, please give your opinion or suggestion:			
4.3 In scenarios where a no-treatment comparator is appropriate, a sequential design might be preferable.			
→If you choose to partially agree or disagree, please give your opinion or suggestion:			

© 2026 Ren Y et al. *JAMA Network Open*.

4.4 Treatment strategies should be clearly categorized as:<br>• Point treatment strategies: Intervention at baseline with predefined exposure windows.<br>• Sustained treatment strategies: Interventions at several times across individuals, requiring methods to account for time-varying confounding. In dynamic regimes, defining treatment status of patients relying on post-baseline characteristics which is affected by prior treatment may induce complex time-varying confounding. The post-baseline characteristics, which also refer to time-varying confounders, would mediate the effect of prior treatment and confounding the effect of current treatment. in such circumstance, simply adjustment for the confounders would cut their mediation effects. Specific strategies such as G-methods, reinforment learning should be applied.			
→If you choose to partially agree or disagree, please give your opinion or suggestion:			
**5. Assignment Procedures**			
5.1 We recommend clearly describing the analytic approach used to emulate random assignment.			
→If you choose to partially agree or disagree, please give your opinion or suggestion:			
5.2 Depending on the type of treatment strategy, different methods may be appropriate:<br>• For effect of treatment assigned at baseline, we recommend the use of standardization, propensity score-based methods (e.g., matching, inverse probability weighting), and doubly robust estimators to adjust for potential baseline confounding.<br>• For effect of sustained treatment, we recommend causal inference methods (e.g., marginal structural model, G-methods, time-dependent propensity score matching) to adjust for time-dependent confounding.<br>Regardless of the type of treatment strategy, we recommend the cloning method to prevent immortal time distortion.			
→If you choose to partially agree or disagree, please give your opinion or suggestion:			
**6. Causal Contrast**			
6.1 It is recommended that the target causal contrast—such as an intention-to-treat (ITT) or per-protocol (PP) effect—be explicitly specified and appropriately justified, based on the research question and clinical context.			

© 2026 Ren Y et al. *JAMA Network Open*.

→If you choose to partially agree or disagree, please give your opinion or suggestion:			
6.2 In general, we suggest the following principles for selecting causal contrast:<br>• ITT effects are generally preferable when evaluating treatment effects assigned at baseline, as they align with the initial assignment strategy and preserve comparability.<br>• Per-protocol effects may be more appropriate when the interest lies in sustained treatment effects, particularly in studies with treatment switching, non-adherence, or long-term exposure dynamics. However, if a substantial number of participants are excluded or censored due to these factors (e.g., treatment switching or non-adherence), per-protocol effects may no longer be appropriate.			
→If you choose to partially agree or disagree, please give your opinion or suggestion:			
6.3 It is advisable to specify primary/secondary causal contrast.			
→If you choose to partially agree or disagree, please give your opinion or suggestion:			
**7. Statistical Analysis**			
7.1 It is recommended that statistical methods be aligned with the defined causal contrast (e.g., intention-to-treat [ITT] vs. per-protocol [PP]).<br>• For ITT analyses, standard regression or weighted models may be appropriate, as they focus on baseline treatment assignment.<br>• For PP analyses, particularly in the presence of time-varying treatments or non-adherence, more advanced methods such as marginal structural models (MSMs) with appropriate weights are encouraged.			
→If you choose to partially agree or disagree, please give your opinion or suggestion:			
7.2 When estimating the PP effect, we recommend using inverse probability of treatment weights to address the selection bias due to non-adherence to the treatment assigned at the baseline.			
→If you choose to partially agree or disagree, please give your opinion or suggestion:			

© 2026 Ren Y et al. *JAMA Network Open*.

7.3 To address selection bias from loss to follow-up in observational time-to-event analyses, post-baseline adjustments (e.g., inverse probability of censoring weights) may be needed to estimate both ITT and PP effects.			
→If you choose to partially agree or disagree, please give your opinion or suggestion:			
7.4 To address potential biases and increase robustness, we recommend:<br>• Directed Acyclic Graphs (DAGs) to guide confounding identification.<br>• Exploring the potential influence of unmeasured confounding using tools such as E-values, instrumental variables, and negative/positive controls.<br>• Sensitivity analyses to test robustness under varying definitions of participants, treatment, outcomes, time zero, causal contrasts and statistical methods.			
If you choose to partially agree or disagree, please give your opinion or suggestion:			
7.5 It is advisable to specify primary/secondary analysis. The statistical analysis plan should prospectively designate whether ITT or PP analysis will serve as the primary analysis, while clearly articulating the complementary role of the alternative approach for secondary/sensitivity analyses.			
→If you choose to partially agree or disagree, please give your opinion or suggestion:			

Do you have any other additional comments or suggestions? If so, please fill in the blanks below. (Not required):

End，Thank you!

© 2026 Ren Y et al. *JAMA Network Open*.

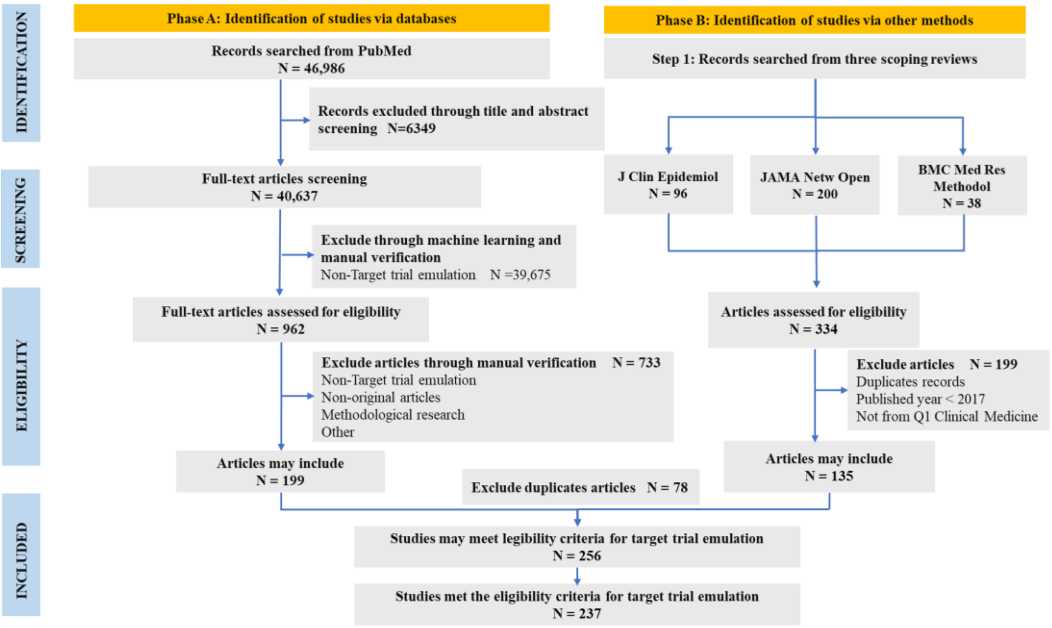

eFigure 1. Flow chart of study selection

© 2026 Ren Y et al. *JAMA Network Open*.

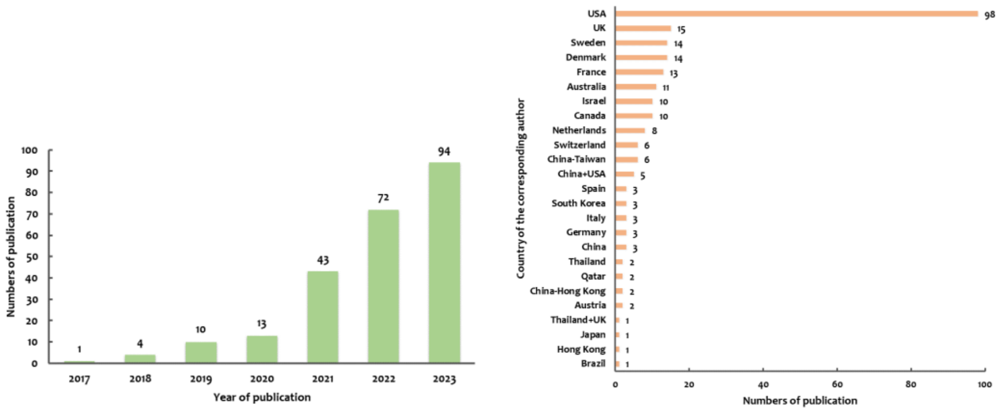

eFigure 2. The trend of TTE studies from 2017 to 2023 and the distribution of corresponding author country

© 2026 Ren Y et al. *JAMA Network Open*.

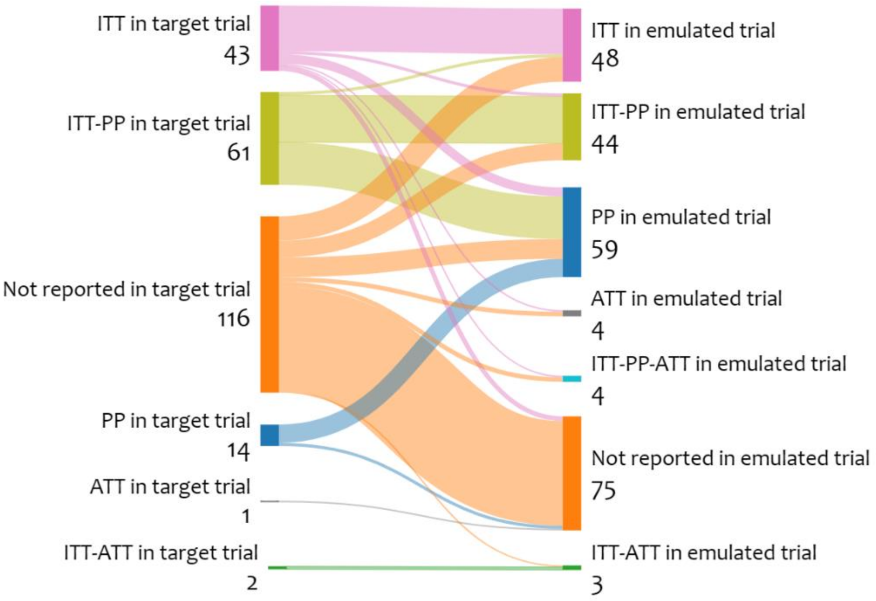

eFigure 3. The causal contrast declared in the target trials vs. in the emulated trials. (ITT: intention-to-treat, PP: per-protocol, ATT: As-treated)

© 2026 Ren Y et al. *JAMA Network Open*.

eTable 1. General characteristics of included TTE studies

Characteristics	All (N, %)
**Study design**	
Prospective cohort study	20(8.5%)
Retrospective cohort study	207(86.4%)
Cohort and case-control	1 (0.9%)
Not reported	*9(4.3%)*
**Data source**	
Registry	*63(26.6%)*
Administrative claims database	*78(32.9%)*
Electronic Medical Records	*118(49.8%)*
Traditional epidemiological survey	*46(19.4%)*
Surveillance database	*50(21.1%)*
Trial data	*8(3.4%)*
**The coverage of the data source**	
One center or hospital	*12(5.1%)*
Regional center	*34(14.3%)*
National center	*175(73.8%)*
International center	*16(6.8%)*
**Sample size, median (IQR)**	
Sample eligible	*15993(3282, 115007)*
Sample analyzed	*11200(2381, 106112)*
**Methodologist involved**	*193(81.4%)*
**Source of funding** [a]	
Government funding	*129(54.4%)*
Private for profit	*29(12.2%)*
Private not for profit	*114(48.1%)*
No funding	*16(6.8%)*
Not reported	*11(4.6%)*

[a] some studies have more than one financial support.

© 2026 Ren Y et al. *JAMA Network Open*.

eTable 2. The methodological characteristics for implementing a TTE study in the included TTE studies

Categories	Variable	All (N, %)
**TTE reporting**	**Did the study report following any methodological guideline?**	
	STROBE	55(23.2%)
	RECORD	5(2.1%)
	ISPOR	5(2.1%)
	Other	5(2.1%)
	Not reported	167(70.5%)
	**How the protocol of the emulated target trial was reported?**	
	TTE framework tabular form	112(47.3%)
	TTE framework txt form	22(9.3%)
	Text form not following TTE framework	103(43.5%)
	**What components of target trial emulation were reported?**	
	Eligibility criteria	232(97.9%)
	Treatment strategies	223(94.1%)
	Assignment procedures	147(62.0%)
	Outcome(s)	232(97.9%)
	Follow-up	207(87.3%)
	Causal contrast of interest	152(64.1%)
	Statistical methods	226(95.4%)
**Eligibility criteria**	**Whether the eligibility criteria in emulated trial are same as those in target trial**	
	Yes	101(42.6%)
	No	37(15.6%)
	Not reported	99(41.8%)
	**Whether the eligibility criteria are based only on values that are available at baseline, never on post-baseline information used to define eligibility**	
	Yes	201(84.8%)
	No	36(15.2%)
	**Whether the study specified eligibility patients at multiple time**	
	Yes	35(14.8%)
	No	202(85.2%)

© 2026 Ren Y et al. *JAMA Network Open*.

	**Whether a flow chart is used to describe who is eligible for TTE?**	
	Yes	*195(82.3%)*
	No	*42(17.7%)*
**Treatment strategies**	**Whether the treatment strategies in emulated trial are same as those in target trial**	
	Yes	*114(48.1%)*
	No	*20(8.4%)*
	Not reported	*103(43.5%)*
	**Whether the treatment strategies consider the active comparator**	
	Yes	*144(60.8%)*
	No	*93(39.2%)*
	**Which design was used in treatment strategies?**	
	Prevalent user design	*4(1.7%)*
	New user design	*71(30.0%)*
	Prevalent/modified new user design	*3(1.3%)*
	Not applicable	*91(38.4%)*
	Not specified	*68(28.7%)*
**Assignment procedures**	**Did the study emulate random assignment procedures?**	
	Yes	*158(66.7%)*
	No	*79(33.3%)*
	**If yes, what methods did the study use to emulate random assignment?**	
	Adjustment (e.g., via IP weighting)	*113(71.5%)*
	Clone	*29(18.4%)*
	Other	*12(7.6%)*
	No methods described	*4(2.5%)*
**Follow-up**	**Whether a design diagram is reported to summarize visually the longitudinal follow-up design aspects of a study?**	
	Yes	*40(16.9%)*
	No	*197(83.1%)*
**Outcome(s)**	**Whether the outcome in emulated trial is same as those in target trial**	
	Yes	*139(58.6%)*
	No	*10(4.2%)*

© 2026 Ren Y et al. *JAMA Network Open*.

	Not reported	*88(37.1%)*
	**Did the author measure the quality of the outcome?**	
	Yes	*37(15.6%)*
	No	*200(84.4%)*
**Causal contrast of interest**	**Did the authors explicitly state their causal contrast of interest?**	
	Yes	*161(67.9%)*
	No	*76(32.1%)*
	**What was the declared or inferred causal contrast in the target trial?**	
	ITT (intention-to-treat effect)	*106(65.8%)*
	PP (per protocol effect)	*75(46.6%)*
	As-treated	*3(1.9%)*
	**What was the declared or inferred causal contrast in the emulated trial?**	
	ITT (intention-to-treat effect)	*98(60.9%)*
	PP (per protocol effect)	*107(66.5%)*
	As-treated	*11(6.8%)*
**Statistical methods**	**Analytic and causal assumptions were stated in the study?**	
	Exchangeability given selected confounders	*151(63.7%)*
	Positivity	*88(37.1%)*
	Consistency	*13(5.5%)*
	Statistical assumptions	*98(41.4%)*
	Not reported	*54(22.8%)*
	**Whether used the statistical method to assess the potential influence of unmeasured confounders?**	
	Yes	*73(30.8%)*
	No	*164(69.2%)*
	**Was sensitivity analysis used to check the robustness of the results?**	
	Yes	*185(78.1%)*
	No	*52(21.9%)*

© 2026 Ren Y et al. *JAMA Network Open*.

eAppendix 5. A Structured Framework for Designing Target Trials

Step 1: Define the causal questions and intervention effect of interest.

A valid TTE must begin with a well-defined causal question concerning the effect of an intervention.

Step 2: Identify the application scenario

It is recommended to begin by clearly identifying the context to which the target trial emulation (TTE) will be applied. TTE application scenarios can generally be categorized into the following three types:

- **Scenario 1:** Replicating or predicting findings from existing randomized controlled trials (RCTs) in real-world settings.
- **Scenario 2:** Extending the evidence base, such as including broader or underrepresented populations, evaluating rare or long-term outcomes, or assessing treatment-switching strategies.
- **Scenario 3:** Setting where RCTs are infeasible due to ethical or logistical constraints.

Accurate classification of the scenario is encouraged, as it informs subsequent design decisions and ensures the appropriateness of the emulation approach.

Step 3: Specify the PICO

Researchers are advised to explicitly define the PICO (Population, Intervention, Comparator, and Outcome) of the hypothetical target trial. A well-specified PICO framework facilitates alignment between the research objective and study design, and enhances clarity and reproducibility.

Step 4: Review existing or similar RCTs to guide trial design:

Where applicable, it is suggested to consult existing RCTs to guide the development of the target trial protocol:

- **For Scenario 1,** emulation should be based on a matched RCT, allowing for comparison or prediction in a real-world population.
- **For Scenario 2**, similar RCTs may be used to inform the plausibility of key design elements in the hypothetical trial.
- **For Scenario 3**, when no RCTs are available, the hypothetical trial should be justified based on clinical rationale, biological plausibility, or expert consensus.

Such review enhances the credibility of the target trial and supports the emulation with precedent evidence where available.

© 2026 Ren Y et al. *JAMA Network Open*.

Step 5: Develop a transparent protocol

It is strongly recommended to develop a clear and detailed protocol that includes:

- Eligibility criteria
- Treatment strategies (definitions of interventions and comparator)
- Assignment procedures
- Follow-up strategies
- Outcome definitions
- Specification of the causal contrast of interest
- Statistical analysis plan

A transparent protocol ensures methodological consistency and supports evaluation of the internal validity and replicability of the emulated trial.

Consider a Dual-Protocol Design to Support Scenario 2 Applications

For Scenario 2, where the research objective extends beyond the scope of existing randomized controlled trials (RCTs)—such as evaluating new outcomes (e.g., safety), broader populations, or longer follow-up periods—it is recommended to develop two related target trial protocols to strengthen methodological transparency and internal consistency, which have been introduced in previous study.

We recommend the following two-step protocol strategy:

• Protocol A (Validation Trial Protocol): design a target trial that emulates a well-conducted existing RCT (e.g., one focused on efficacy). This emulation should closely align with the original RCT in terms of population, intervention, comparator, outcomes, and follow-up duration. The aim is to verify that the emulated design yields comparable effect estimates—at minimum, demonstrating consistency in the direction of effect. This serves as a methodological benchmark, ensuring that key trial elements are accurately operationalized in the real-world dataset.

• Protocol B (Extension Trial Protocol): based on the validated design of Protocol A, construct a second target trial addressing the actual research question of interest (e.g., assessing safety outcomes or evaluating effects in underrepresented populations). This protocol may appropriately modify certain parameters such as outcomes, eligibility criteria, or follow-up time, while retaining the structural integrity confirmed in Protocol A. The rationale for these modifications should be explicitly stated and grounded in both clinical

reasoning and the findings of Protocol A.

This dual-protocol approach is especially valuable when extending beyond the evidence base of existing RCTs. It supports transparent justification of design choices, enhances reproducibility, and strengthens the interpretability of causal inference derived from observational data.

Part II: Recommendations for implementing core methodological components in TTE

1. Eligibility Criteria

1.1 We recommend that eligibility criteria be defined strictly based on information available at baseline (i.e., prior to treatment initiation) to avoid post-baseline selection bias.

1.2 In situations where patients may meet eligibility criteria at multiple time points, we recommend considering a sequential trial emulation approach. This can improve statistical power while maintaining alignment with the target trial framework.

2. Follow-up and Time Zero Definition

2.1 We recommend that time zero (i.e., the start of follow-up) be clearly and explicitly defined, with alignment between eligibility assessment and treatment assignment. Misalignment among these elements may occur and could introduce biases such as selection bias, prevalent user bias, and immortal time bias. Appropriate methodological approaches should be employed to address these potential biases.

2.2 It is recommended to consider the use of look-back period before time zero to assess prior medical history, baseline covariates, and registration duration. This approach may help ensure more accurate adjustment for confounding and more appropriate specification of inclusion criteria.

2.3 To improve clarity and reproducibility, we suggest using a graphical timeline or design diagram to visually illustrate the structure of follow-up, especially when eligibility and treatment occur asynchronously.

3. Outcome Definition and Measurement

3.1 It is advisable to prespecify the outcome(s) of interest, including their clinical definitions, time frames, and relevance to the causal question.

3.2 Data sources used for outcome identification should be clearly documented (e.g.,

© 2026 Ren Y et al. *JAMA Network Open*.

electronic health records, claims data), along with the validation methods and reliability metrics.

3.3 We encourage transparent reporting of the methods used to measure and verify outcomes, including any algorithms or clinical adjudication processes applied.

3.4 It is advisable to specify primary/secondary outcome(s) in the case of multiple outcomes

4.Treatment strategies

4.1 When feasible, an active-comparator new-user design is recommended to assess the real-world safety and effectiveness of medications; only patients who are the first dispensation recorded at time zero and who are naive to both treatments in the previous grace period are included. This design is largely due to the baseline washout period (mimicking a clinical trial) and using an active comparator to reduce confounding by indication. However, this design does not fully eliminate indication bias, as treatment choices are still influenced by disease severity.

4.2 In modified/prevalent new-user designs—which include both patients previously exposed to the comparator and those who are treatment-naïve—this design may be particularly useful in drug safety evaluations when comparing a newly introduced treatment with a long-established comparator.

4.3 In scenarios where a no-treatment comparator is appropriate, a sequential design might be preferable.

4.4 Treatment strategies should be clearly categorized as:

- Point treatment strategies: Intervention at baseline with predefined exposure windows.
- Sustained treatment strategies: Interventions at several times across individuals, requiring methods to account for time-varying confounding. In dynamic regimes, defining treatment status of patients relying on post-baseline characteristics which is affected by prior treatment may induce complex time-varying confounding. The post-baseline characteristics, which also refer to time-varying confounders, would mediate the effect of prior treatment and confounding the effect of current treatment. in such circumstance, simply adjustment for the confounders would cut their mediation effects. Specific strategies such as G-methods, reinforment learning should be applied.

© 2026 Ren Y et al. *JAMA Network Open*.

5. Assignment Procedures

5.1 We recommend clearly describing the analytic approach used to emulate random assignment.

5.2 Depending on the type of treatment strategy, different methods may be appropriate:

- For effect of treatment assigned at baseline, we recommend the use of standardization and propensity score-based methods (e.g., matching, inverse probability weighting) to adjust for potential baseline confounding.
- For effect of sustained treatment, we recommend causal inference methods (e.g., marginal structural model, G-methods, time-dependent propensity score matching) to adjust for time-dependent confounding.

When treatment strategies that cannot be distinguished at the start of follow-up (e.g., the presence of a grace period or when treatment must be sustained for a minimum duration before classification into the exposed group), the cloning method may be applied to prevent immortal time distortion.

6. Causal Contrast

6.1 It is recommended that the target causal contrast—such as an intention-to-treat (ITT) or per-protocol (PP) effect—be explicitly specified and appropriately justified, based on the research question and clinical context.

6.2 In general, we suggest the following principles for selecting causal contrast:

- ITT effects are generally preferable when evaluating treatment effects assigned at baseline, as they align with the initial assignment strategy and preserve comparability.
- Per-protocol effects may be more appropriate when the interest lies in sustained treatment effects, particularly in studies with treatment switching, non-adherence, or long-term exposure dynamics. However, if a substantial number of participants are excluded or censored due to these factors (e.g., treatment switching or non-adherence), per-protocol effects may no longer be appropriate.

6.3 It is advisable to specify primary/secondary causal contrast.

7. Statistical Analysis

7.1 It is recommended that statistical methods should be aligned with the defined causal contrast (e.g., intention-to-treat [ITT] vs. per-protocol [PP]).

- For ITT analyses, standard regression or weighted models may be appropriate, as they focus on baseline treatment assignment.
- For PP analyses, particularly in the presence of time-varying treatments or non-

© 2026 Ren Y et al. *JAMA Network Open*.

adherence, more advanced methods such as marginal structural models (MSMs) with appropriate weights are encouraged.

7.2 When estimating the PP effect, we recommend using inverse probability of treatment weights to address the selection bias due to non-adherence to the treatment assigned at the baseline.

7.3 To address selection bias from loss to follow-up in observational time-to-event analyses, post-baseline adjustments (e.g., inverse probability of censoring weights) may be needed to estimate both ITT and PP effects.

7.4 To address potential biases and increase robustness, we recommend:

- Directed Acyclic Graphs (DAGs) to guide confounding identification.
- Exploring the potential influence of unmeasured confounding using tools such as E-values, instrumental variables, and negative/positive controls.
- Sensitivity analyses to test robustness under varying definitions of participants, treatment, outcomes, time zero, causal contrasts and statistical methods.

7.5 It is advisable to specify primary/secondary analysis. The statistical analysis plan should prospectively designate whether ITT or PP analysis will serve as the primary analysis, while clearly articulating the complementary role of the alternative approach for secondary/sensitivity analyses.

7.6 For dynamic treatment strategies, we recommend employing clone-censor-weight approach for time-dependent confounding and immortal time bias, while employing causal inference methods like G-methods or doubly robust estimators to quantify treatment effects.

© 2026 Ren Y et al. *JAMA Network Open*.
