# Supplementary material for: Design and Implementation of Observational Studies Emulating a Target Trial
Source: JAMA Netw Open. 2026 Feb 19;9(2):e2558262. doi: 10.1001/jamanetworkopen.2025.58262 (PMC12921535; doi:10.1001/jamanetworkopen.2025.58262)
Supplement: Supplement 2. — Data Sharing Statement [file jamanetwopen-e2558262-s002.pdf]

Data Sharing Statement

Ren. Design and Implementation of Observational Studies Emulating a Target Trial. *JAMA Netw Open*. Published February 19, 2026. doi:10.1001/jamanetworkopen.2025.58262

Data
Data available: No
